# Structural basis for autoinhibition and its relief of MOB1 in the Hippo pathway

**DOI:** 10.1038/srep28488

**Published:** 2016-06-23

**Authors:** Sun-Yong Kim, Yuka Tachioka, Tomoyuki Mori, Toshio Hakoshima

**Affiliations:** 1 Structural Biology Laboratory, Nara Institute of Science and Technology, 8916-5 Takayama, Ikoma, Nara 630-0192, Japan

## Abstract

MOB1 protein is a key regulator of large tumor suppressor 1/2 (LATS1/2) kinases in the Hippo pathway. MOB1 is present in an autoinhibited form and is activated by MST1/2-mediated phosphorylation, although the precise mechanisms responsible for autoinhibition and activation are unknown due to lack of an autoinhibited MOB1 structure. Here, we report on the crystal structure of full-length MOB1B in the autoinhibited form and a complex between the MOB1B core domain and the N-terminal regulation (NTR) domain of LATS1. The structure of full-length MOB1B shows that the N-terminal extension forms a short β-strand, the S_N_ strand, followed by a long conformationally flexible positively-charged linker and α-helix, the Switch helix, which blocks the LATS1 binding surface of MOB1B. The Switch helix is stabilized by β-sheet formation of the S_N_ strand with the S2 strand of the MOB1 core domain. Phosphorylation of Thr12 and Thr35 residues structurally accelerates dissociation of the Switch helix from the LATS1-binding surface by the “pull-the-string” mechanism, thereby enabling LATS1 binding.

The Hippo pathway is a key signaling cascade that ensures organ size and normal tissue growth by coordinating cell proliferation and differentiation, and has now been recognized as an essential tumor suppressor cascade^1^,^2^,^3^,^4^,^5^,^6^. In mammals, the core pathway components comprise two Ser/Thr protein kinases, mammalian Ste20-like 1 and 2 (MST1/2) kinases, members of the Ste20 group in the germinal center kinase II (GCK-II) subgroup, and large tumor suppressor 1 and 2 (LATS1/2) kinases, members of the AGC protein kinase family, in addition to transcriptional co-activator Yes-associated protein (YAP) and transcriptional co-activator with PDZ-binding motif (TAZ). To exert full kinase activity, MST1/2 and LATS1/2 requires coactivators, the scaffolding proteins Salvador homolog 1 (SAV1) and Mps One binder 1 (MOB1), respectively. In the active state of the Hippo pathway, Sav1-bound MST1/2 kinases phosphorylate the C-terminal hydrophobic motif (HM) of LATS1/2 and the N-terminal region of MOB1 proteins^7^,^8^,^9^. Phosphorylated MOB1 protein is switched ON to increase the binding affinity to LATS1/2 and this binding accelerates auto-phosphorylation of the activation loop, A-loop (also referred to as activation segment or T-loop)^10^, of LATS1/2 kinases for full activation. Activated MOB1-bound LATS1/2 phosphorylates YAP to block its nuclear import and results in inhibition of its transcriptional programs. In addition to this canonical pathway, mammalian cells possess a number of regulatory side branches for the Hippo pathway^11^,^12^,^13^,^14^,^15^, whereas MOB1 is indispensable for LATS1/2 kinase activation, as shown in yeast and Drosophila^16^,^17^,^18^,^19^. The importance of MOBs in protein kinase activation was first exemplified by yeast nuclear Dbf2-related 1 and 2 (NDR1/2) kinases in the regulation of the mitotic exit network (MEN)^20^ and Ace2p activity and cellular morphogenesis (RAM)^21^,^22^. Thus, MOB proteins act as a pivotal molecular switch both in the Hippo and MEN/RAM pathways.

In mammals, at least six different MOB proteins are encoded by independent genes, and two closely-related (95% sequence identity) members, MOB1A and MOB1B, play redundant biological roles as co-activators of LATS1/2 and NDR1/2 kinases (LATS/NDR kinases)^8^,^19^,^23^. The phosphorylation of MOB1 occurs at two threonine residues, Thr12 and Thr35, located at the N-terminal extension out of the C-terminal globular MOB core domain (Fig. 1a), and is required for binding to LATS/NDR kinases^8^,^23^. Unfortunately, the N-terminal extension of MOB1 is easily degraded during protein purification and all reported MOB1 structures have been based on N-terminal truncated forms^24^,^25^,^26^,^27^,^28^. Lack of a full-length MOB1 structure in the autoinhibited form is hampering efforts to fully understand the mechanism by which Thr12/Thr35-phosphorylation activates MOB1. Insights into this area are crucial, since the phosphorylation of human MOB1 appears to be important in cell proliferation and the suppression of carcinoma development^8^,^29^. Biochemical evidence suggests that phosphorylated MOB1 binds the highly conserved N-terminal regulatory (NTR) region of LATS/NDR kinases^30^,^31^,^32^,^33^,^34^,^35^. The NTR region is located at the immediate front of the catalytic domain (Fig. 1a)^7^,^8^,^9^,^31^ and is essential for kinase activity^8^,^20^,^35^. Recently, two X-ray structures of MOB1 bound to LATS/NDR kinases have been reported: one is a low resolution (3.3 Å–4.5 Å) structure of yeast truncated Mob2p bound to the second NDR-like kinase Cbk1p in the RAM pathway, the Mob2p-Cbk1p complex^36^ and the other structure is that of human phosphorylated MOB1A bound to the NTR domain of LATS1, the pMOB1A-LATS1 structure^37^. However, we found that the structures of the NTR domains and their binding modes in these recently-reported complexes display sharp discrepancies, which should be solved by additional experiments.

Here we report the results of structural and biochemical studies of full-length mouse MOB1B in the autoinhibited form and that of the MOB core domain bound to the NTR region of LATS1 (hereafter referred to as the MOB1B-LATS1 complex). Our structure of full-length MOB1B reveals that the N-terminal extension comprising a positively-charged region followed by Switch α-helix binds directly to the LATS1-binding surface to block LATS1 binding and this blocking is stabilized by β-sheet formation between S_N_ strand of the N-terminal extension and S2 strand of the core domain. Our structure suggests that phosphorylation of Thr12/Thr35 causes relief of autoinhibition by inducing destabilization of Switch helix binding to the LATS1-binding site. This autoinhibited mechanism differs from the recently-proposed model in which the N-terminal FxFx5KxF motif occupies part of the LATS1-binding surface^37^. Our autoinhibited form of full-length MOB1B suggests that N-terminal truncated MOB1B should be capable of binding LATS1. We confirmed this hypothesis by determining the complex structure between N-terminal truncated MOB1B and the NTR domain of LATS1, the MOB1B-LATS1 complex. We found that our structure is essentially the same as the recently reported complex structure between phosphorylated MOB1A and the NTR domain of LATS1, the pMOB1A-LATS1 complex^37^, which established the bihelical V-shaped structure of the NTR domain and its specific interactions with conserved residues located at the LATS1-binding surface. However, the binding mode found in these complex structures differs from that described for the recently-reported structures of the yeast Mob2p-Cbk1p complexes^36^. The MOB1B-LATS1 and pMOB1A-LATS1 structures are consistent with our mutational data and relevant experimental data from the literature, in contrast to those of the Mob2p-Cbk1p complexes. We have concluded that the structural discrepancy is not due simply to intrinsic differences between mouse/human MOB1B and yeast Mob2p. Our study enhances our understanding of the molecular mechanisms involved in the regulation of the Hippo pathway.

## Results

### The overall structure of full length MOB1B

**Figure 1:**
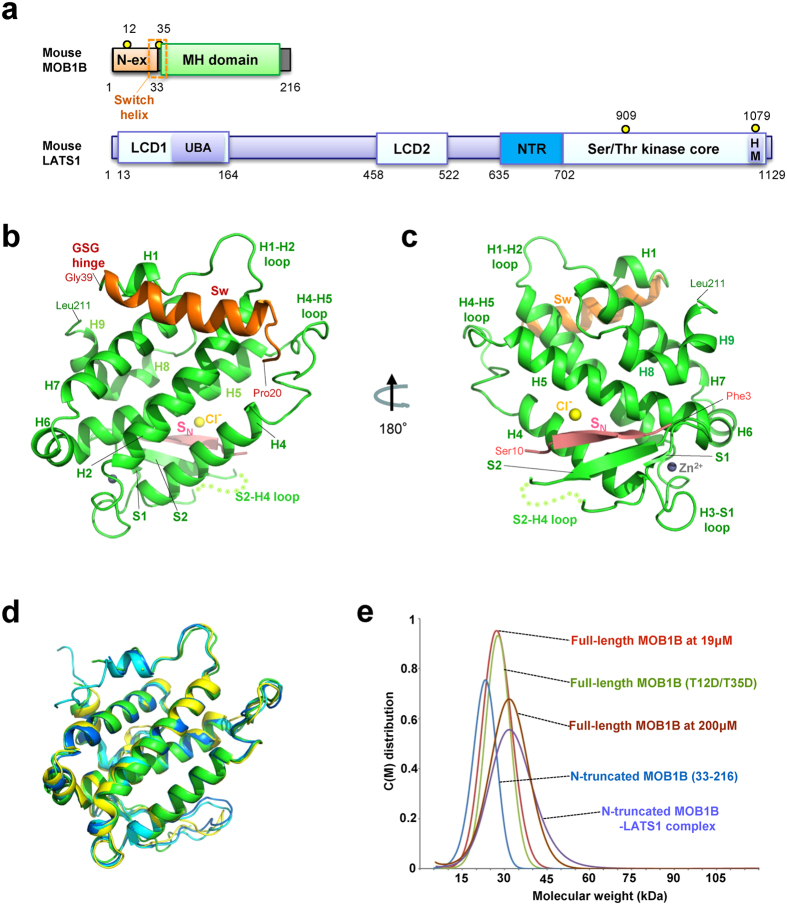
The full-length MOB1B structure shows the autoinhibited form of MOB1. (**a**) Schematic representation of domain organization of MOB1 and LATS1^7^,^8^,^9^,^31^. (**b**) The structure of full-length MOB1B in this study. The front view of MOB1B showing the Switch helix (Sw in orange) bound to the α-helices (H1, H2 and H7) and the H4-H5 loop of the core domain (green). The S_N_ strand (pink) forms a β-sheet with the S2 strand. On the current electron density map, five residues (101-105, indicaed by a dotted line) in the S2-H4 loop were not modeled due to poor density. The nomenclature employed for secondary structures follows that utilized in the first MOB1 structure, human MOB1A^24^. (**c**) As in **b**, but rotated around the vertical axis to show the backside of the molecule. One zinc ion (gray ball) is located in the space between the H3-S1 loop and the H5 helix. (**d**) Structural comparison of full-length mouse MOB1B and related MOB structures. The core domain of full-length mouse MOB1B (green) was superimposed with human MOB1A (cyan), yeast Mob1p (yellow), and phosphopeptide-bound human MOB1A (blue). The root mean square (r.m.s.) deviation on overlay with mouse MOB1B is 0.9 Å (human MOB1A), 2.1 Å (*Xenopus* MOB1), and 1.2 Å (yeast Mob1p). (**e**) Sedimentation velocity ultracentrifugation analyses of full-length, N-terminal 32-residue truncated, and mutant (T12D/T35D) MOB1B show that all of these molecules exist as a monomer in solution. All samples contained 19 μM protein, other than the high-concentration (200 μM) sample of full-length MOB1B. The calculated molecular mass of each sample is 26.9 kDa for full-length MOB1B (19 μM), 27.2 kDa for mutant full-length MOB1B (T12D/35D), 22.2 kDa for N-terminal truncated MOB1B, 31.8 kDa for the N-terminal truncated MOB1B-LATS1 complex, and 30.6 kDa for 200 μM full-length MOB1B.

As reported previously for human and Xenopus MOB1^24^,^25^, the N-terminal extension of mouse MOB1B was also protease sensitive. However, we successfully purified full-length MOB1B by low-temperature expression (see experimental procedures) and quick purification without degradation (Supplementary Fig. 1), and determined the three-dimensional structure at 2.2 Å resolution (Fig. 1b,c) (Table 1). The asymmetric unit contains four independent molecules (Supplementary Fig. 2a), which display no large conformational changes (Supplementary Fig. 2b). The overall structure adopts a globular shape comprising the MOB core domain made of nine α-helices (H1-H9) with the N-terminal extension, which forms a β-strand (the S_N_ strand, residues 5-9) at the N-terminal segment of FLFGSRSS (residues 3-10) and a newly formed 4-turn α-helix (residues 24-38, hereafter referred to as the Switch helix), whereas the 10-residue linker between the S_N_ strand and the Switvch helix were poorly defined on the current map. Notablly, the S_N_ strand forms a β-sheet with the S2 strand (residues 95-98) (Fig. 1c).

The MOB core domain composed of nine α-helices H1-H9 and two small β-strands (S1 and S2), which form a hairpin-like structure (Fig. 1b and Supplementary Fig. 3). The nomenclature employed for secondary structures follows that utilized for the first MOB1 structure, human MOB1A^24^. One zinc ion is coordinated by two cysteine (Cys79 and Cys84) and two histidine (His161 and His166) residues and linked to the H3-S1 loop and the C-terminal part of the H5 helix as found in the structures of other MOB proteins^24^,^25^,^26^,^27^,^28^ (Fig. 1c and Supplementary Fig. 2c). Thus, the core domain of MOB1B displays essentially the same structure as those of the N-terminal truncated free forms of human MOB1A^24^, *Xenopus* MOB1^25^, and yeast Mob1p^26^ (Fig. 1d).

### MOB1B exists as a monomer in solution

Our analytical ultracentrifugation (AUC) experiments of full-length MOB1B, its phosphomimic (T12D/T35D) double-mutant and the N-terminal 32-residue truncated form show a single monomeric peak at ~30 kDa, indicating that MOB1B exits as a monomer in solution (Fig. 1e). Yeast Mob1p, however, was proposed to form a dimer in solution, based on the light-scattering data and crystal packing showing part of its N-terminal residues making intermolecular contacts with an asymmetric mate^26^. This discrepancy could be attributed to absence of the extremely long N-terminal extension (~140 residues) of yeast Mob1p. The helical region (H0, residues 126-139) of yeast Mob1p partially overlaps with the Switch helix, although the sequence homology is poor (Supplementary Fig. 2). Another reason for the discrepancy may be the higher protein concentration (~20 mg/ml, ~700 μM) utilized in the light-scattering experiments compared to that employed in our experiments (19 μM). We confirmed a monomer in a higher concentration (200 μM) of full-length MOB1B (Fig. 1e) and thus concluded that MOB1B exists as a monomer in solution.

### Switch helix formation and binding to the MOB core domain

**Figure 2:**
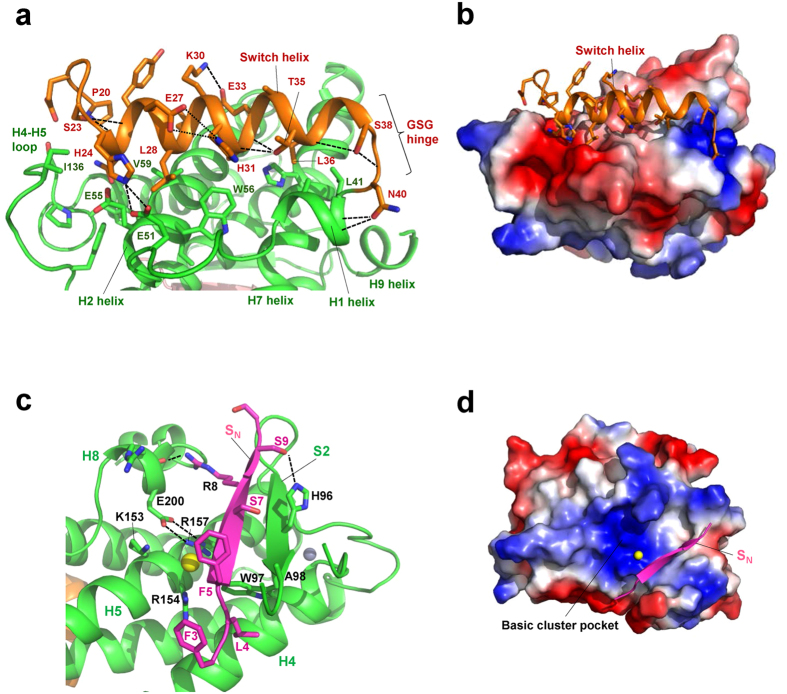
The N-terminal extension binds the core domain by forming the S0 strand and the Switch helix. (**a**) Close-up view of the Switch helix bound to the MOB core domain. The Switch helix (orange) binds the MOB core domain (green). The Switch helix is stabilized by intra-helical salt bridges/ionic hydrogen bonds (broken lines) and polar and nonpolar interactions with the core domain. Thr35 is located at the interface between the Switch helix and the core domain and makes hydrogen bonds to the main-chain carbonyl groups of Ala31 and His32 of the Switch helix. (**b**) Electrostatic surface potential shows the Switch helix bound to the negatively-charged (red) and nonpolar-patched molecular surface formed by α-helices H2 and H7 and the H4-H5 loop (see text). (**c**) Close-up view of the peptide-binding site of molecule-A. The S_N_ strand (magenta) binds the peptide-binding site by forming a β-sheet with the S2 strand, and is stabilized by nonpolar contacts of Leu4 with Trp97 and Phe5 with Ala98, and hydrogen bonds of Arg8 to the H8 helix and Ser7 and Ser9 with His96. The peptide binding site is adjacent to the basic cluster pocket formed by Lys153, Arg154 and Arg157 from helices H4 and H5. The bound chlorine anion (yellow ball) is located at the center of the basic residues, which is the phosphate group-binding site in the structure of human MOB1 bound to the Nud1-like phospho-peptide^27^. (**d**) The electrostatic surface potential of the basic cluster pocket with the S_N_ strand and the bound chlorine anion (yellow ball).

The Switch helix is stabilized by forming N-terminal capping with Ser23 and intra-helical salt bridges (Glu27-His31 and Lys30-Glu33) at the helix surface (Fig. 2a). The Switch helix is connected to a Gly-Ser-Gly (37–39) linker, which may be conformationally flexible and responsible for the observed protease susceptibility^24^,^25^,^26^,^28^, and is followed by the H1 helix stabilized by the N-terminal cap of Asn40. The Switch helix-binding site on the MOB core domain is formed by helices H1, H2 and H7 and the H4-H5 loop, and is characterized by a conserved cluster of negative charges with nonpolar patches (Fig. 2b). This site is overlapped with the LATS1 binding surface found in the structures of the pMOB1A-LATS1 complex^34^, the Mob2p-Cbk1p complex^36^ and our MOB1B-LATS1 complex, which are described below in detail, suggesting that the autoinhibition is caused by Switch helix binding, which structurally blocks LATS/NDR binding. This molecular surface corresponds to that previously hypothesized to interact with LATS/NDR kinases^24^,^25^.

The interface between the Switch helix and MOB core domain contains several polar and nonpolar contacts. Positively-charged His24 forms salt bridges with Glu51 and Glu55 and bifurcated hydrogen bonds to Glu51 to lock the Switch helix onto the MOB core domain (Fig. 2a). Nonpolar residues (Leu28, Leu29, Ala32, and Leu36) of the Switch helix contact the nonpolar patches formed by the H1 helix (Leu41), H2 helix (Trp56, Val59) and H4-H5 loop (Ile136). The phosphorylation residue of Switch helix (Thr35) is located at the edge of the interface with the MOB core domain and forms two hydrogen bonds to the helix main chains (Fig. 2a). Therefore, once phosphorylation occurs, phosphorylated Thr35 structurally inhibits binding of the Switch helix to the MOB core domain, which is consistent with previous reports that showed a critical role of Thr35 phosphorylation in LATS2 activation^23^. The structure also implies that the active site of MST1/2 kinases could not access the side chain of Thr35 without dissociation of the Switch helix from the core domain, suggesting that MST1/2 traps the transiently dissociated Switch helix, the latter involved in a dynamic association-dissociation process.

### Peptide-binding site formed by a β-sheet

The N-terminal S_N_ strand forms an antiparallel β-sheet with the S2 strand, which is located at the molecular surface opposite the Switch helix (Fig. 2c). This β-sheet formation is reminiscent of the intermolecular β-sheet formed in the complex between Nud1-like phospho-peptide and human MOB1^27^. In our structure, one chlorine anion binds the basic cluster pocket (formed by Lys153, Arg154 and Arg157 from helices H4 and H5) (Fig. 2d), which corresponds to the phosphate-binding pocket for the Nud1-like phospho-peptide, suggesting that the S2 strand and the basic cluster pocket provide an active site for inter- or intramolecular interactions. Another phosphorylation residue, Thr12, is located at the conformationally flexible part of the N-terminal extension, flanking the C-terminal end of the S_N_ strand, indicating that this residue may be easily accessible to phosphorylation by MST1/2, and contrasts that of Thre35 which is partially buried at the interface between the Switch helix and core domain (Fig. 2a).

### The MOB1 autoinhibition and the “pull-the-string” mechanisms of its relief inferred from a structural comparison between the autoinhibited and relieved forms

**Figure 3:**
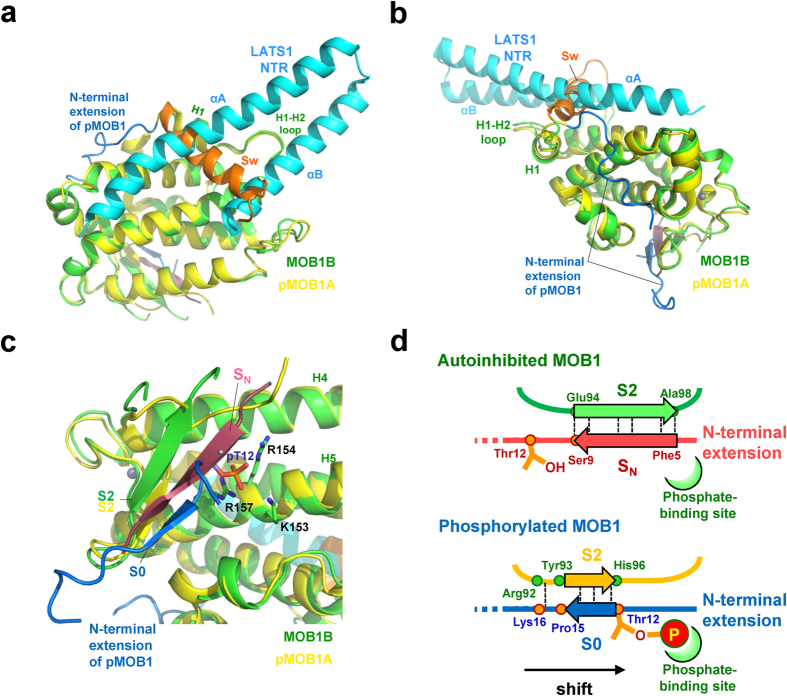
The structural autoinhibition of MOB1 inferred from structural comparison of full-length MOB1B with the pMOB1-LATS1 complex. (**a**) Full-length MOB1B is superimposed on phosphorylated MOB1A of the pMOB1A-LATS1 complex (PDB code 5BRK) with an overall r.m.s. deviation of 0.99 Å. Direct overlap of the Switch helix of our full-length MOB1B and the NTR helices of LATS1 (cyan) bound to phosphorylated MOB1A indicates that the autoinhibition is mediated *via* a structural block of the LATS1-binding site by the Switch helix (Sw, orange). (**b**) As in **a**, but a view from the backside of the LATS1-binding site to show the large conformational shifts of the phosphorylated N-terminal extension (blue). (**c**) Close-up view of the overlay of β-sheets formed by the S_N_/S0-S2 strands of our full-length MOB1B and phosphorylated MOB1A of the pMOB1A-LATS1 complex. The phosphorylated Thr12 residue (pT12, orange) of the N-terminal extension (blue) binds the basic cluster pocket. (**d**) Schematic drawing of the “pull-the-string” mechanism to show the shift of the N-terminal extension by phosphorylation. The phosphorylated Thr12 residue pulls down the peptide chain of the N-terminal extension toward the backside of the MOB1 core domain to bind the phosphate-binding site.

We compared our autoinhibited structure with the recently reported structure of phosphorylated MOB1A in the pMOB1A-LATS1 complex^37^ in an effort to define the structural changes induced by autoinhibition and its relief. The NTR domain (residues 635-702) of LATS1forms a V-shaped bi-helical structure comprising two long α-helices, αA and αB, and superposition of these structures shows direct overlap of the Switch helix with both αA and αB helices of the NTR domain (Fig. 3a), thus defining the conformational autoinhibition of MOB1 binding to LATS1 *via* the Switch helix of the N-terminal extension. No other segments of MOB1 display any direct interference in binding to the NTR domain.

The superimposed structures also shows that the N-terminal extension residues (1-40) display a drastic conformational transition, whereas the H1 helix and following residues forming the core domain only undergo local conformational changes in the S2-H4 loop, which is partially disordered in both structures (Fig. 3b). The H1 helix of the autoinhibited form is followed by a sharp turn of Ser38-Gly39 and the Switch helix. The turn is stabilized by the Ser38 side chain forming hydrogen bonds with the main chains of Thr35 from the H1 helix and Asn40 from the Switch helix (Fig. 2a). This turn is absent in the phosphorylated form and the peptide chain is extended to run through the shallow groove formed by helices H6-H7 and H9 and is followed by a disordered region. The extended structure is stabilized by Ser38 interacting with the phosphate group of Thr35 and a short 3_10_-helix (H0) formed by Leu-Leu-Lys at positions 28–30, which bind the hydrophobic site of the shallow groove. In the autoinhibited form, the Leu-Leu-Lys segment is part of the Switch helix and two Leu residues participate in nonpolar interactions with the H2 helix.

The phosphorylated Thr12 residue of the pMOB1A-LATS1 complex is located close to the S2 strand so that the phosphate group is docked into the basic cluster pocket (Fig. 3c). The N-terminal end residues (1-9) are disordered but the three residues (13-15) that follow pThr12 form the S0 strand, which associates with the S2 strand to form a short antiparallel β-sheet. This S0-S2 β-sheet formation differs from that of the S_N_-S2 β-sheet found in the autoinhibited form in several ways. First, the S_N_ strand of the autoinhibited form is formed by five residues and is longer than the S0 strand. Second, the residues forming the S_N_ strand are located at positions 5–9 (Phe5 to Ser9) and represent a 6-residue shift toward the N-terminal end (Fig. 3d). It is likely that on phosphorylation, the peptide chain of the N-terminal extension is pulled down toward the basic cluster pocket for phosphorylated Thr12 binding, resulting in a frame shift of residues forming the β-strand associated with the S2 strand. This shift should induce dissociation of the Switch helix, which is also accelerated by phosphorylation of Thr35 located at the helix. We refer to this mechanism as the “pull-the-string” mechanism for MOB1 activation.

**Figure 4:**
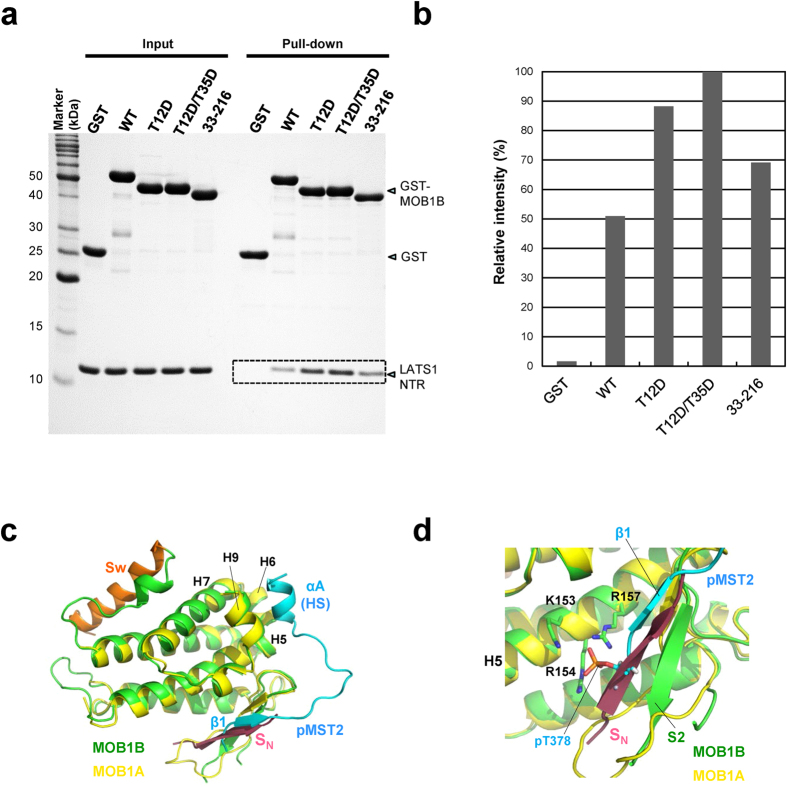
MOB1 autoinhibition by the N-terminal extension and its release by truncation or phosphorylation. (**a**) Pull-down binding assays of the NTR domain of LATS1 with GST-MOB1B. MOB1B proteins are the N-terminal 32-residue truncated form (33–216), the wild type (WT) and double-mutant (T12/35D, phosphormimetic replacement of Thr12 and Thr35 with Asp) full-length forms. Full-length MOB1B exhibits residual binding affinity to the NTR domain of LATS1, consistent with the reported weak binding of full-length MOB1A to LATS1^37^. (**b**) Histogram of LAT1 binding to GST-MOB1B shown in **a**. Band intensities were visualized using LAS-4000 (Fujifilm) and the program ImageGauge (Fujifilm) (see Materials and Methods). The relative intensities were normalized to that of the double phosphomimetic (T12D/T35D) form (100%). (**c**) Our full-length MOB1B superimposed on N-truncated MOB1A of the MOB1A-pMST2 complex (PDB code 5BRM). The backside from the LATS1-binding site is viewed to show the overlap of the S_N_ strand of MOB1B and the β1 strand of pMST2. The hydrophobic sequence (HS) of pMST2 forms the short αA helix that binds helices H5, H6, H7 and H9 of MOB1. The MOB core domains are overlaid with an overall r.m.s. deviation of 0.87 Å. (**d**) Close-up view of the overlay of β-sheets formed by the S_N_-S2 strands of full-length MOB1B and by the β1-S2 strands in the MOB1A-pMST2 complex. The phosphate group (orange) of pT378 of pMST2 binds the basic cluster pocket.

Our structure of the autoinhibited form of MOB1B showing the absence of large conformational changes in the MOB core domain suggests that truncation of the N-terminal extension converts the autoinhibited form to an active form. We tested this idea by pull-down binding assays of the NTR domain of LATS1 with GST-MOB1B. For this experiment, the NTR domain was purified by resolubilization (see Materials and Methods). The N-terminal 32-residue truncation, which removes most of the Switch helix, enhances binding to the NTR domain of LATS1 (Fig. 4a,b). Phosphomimetic substitution of Thr12 and Thr35 with an aspartate residue also induces enhancement of LATS1 binding. Intriguingly, the phosphomimetic forms exhibited stronger affinity than the truncated form, implying that the phosphrylated N-terminal extension contributes to stabilization of the unmasked state. Indeed, a loop region between the H1 helix and the phosphorylated Thr35 residue contact the LATS1 NTR helix (Fig. 3b). The single phosphomimetic substitution of Thr12 activates LATS1 binding, and the double phosphomimetic substitution of both Thr12 and Thr35 induces further activation. Thus, phosphorylation of these two residues should cooperate to relieve autoinhibition by N-terminal extension.

### Structural basis of MOB1 activation by binding to MST1/2

We compared our autoinhibited structure with that of the N-truncated MOB1A bound to the phosphorylated MST2 peptide from a linker between the MST2 kinase and SARAH domains, hereafter referred to as the MOB1A-pMST2 complex^27^ (Fig. 4c). The MST2 peptide has a phosphorylated threonine residue (pThr378) located at the N-terminal region, which forms a β1 strand associated with the S2 strand of MOB1A, with the phosphate group of pThr378 docked into the basic cluster pocket (Fig. 4d). This binding mode resembles that of the N-terminal extension of phosphorylated MOB1A, which forms a short β-sheet between the S0 and S2 strands with pThr12 docked into the basic cluster pocket (Fig. 3c). Furthermore, the β-sheet formed between the β1 strand of MST2 and the S2 strand of MOB1A is overlapped with the β-sheet of the S_N_-S2 strands of autoinhibited MOB1B. This overlap induces competition between the β1 strand of MST2 and the S_N_ strand of autoinhibited MOB1, and should contribute to MOB1 activation by MST2 binding as previously observed^27^. In the MOB1A-pMST2 complex, the MST2 peptide forms a short α-helix (αA), which binds the hydrophobic sequence (HS)-binding site. Since this binding site is overlapped with the binding site for the H0 helix of the phosphorylated N-terminal extension in the pMOB1A-LATS1 complex, activation of MOB1 by binding to pMST2 *via* competition for binding to this overlapped binding site was proposed^27^. However, in our autoinhibited form, we did not observe binding of any region of the N-terminal extension to the HS-binding site.

### Contribution of the S_N_ strand to MOB1 autoinhibition

**Figure 5:**
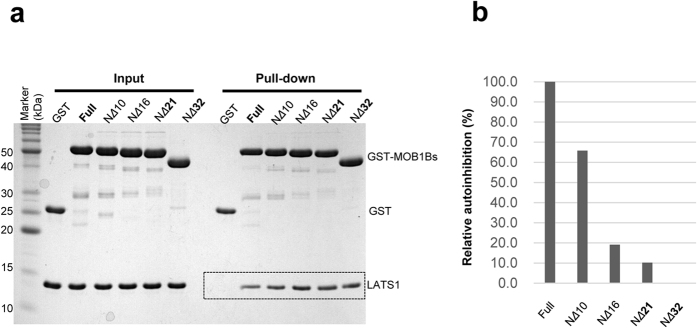
The S_N_ strand of the N-terminal extension of MOB1B contributes to stabilization of the autoinhibited form. (**a**) Pull-down binding assays of the NTR domain of LATS1 with GST-N-terminal truncated MOB1B. MOB1B proteins are the N-terminal 10, 16, 21, and 32-residue truncated forms, N*Δ*10, N*Δ*16, N*Δ*21 and N*Δ*32, respectively, with the full-length form (Full). Full-length MOB1B exhibits residual binding affinity to the NTR domain of LATS1 (see Fig. 4a). (**b**) Histogram of relative autoinhibitory effects on LAT1 binding to that of GST-N-truncated *Δ*32 MOB1B shown in **a**. Band intensities were visualized using LAS-4000 (Fujifilm) and the program ImageGauge (Fujifilm) (see Materials and Methods). The relative autoinhibition were normalized to that of the wild-type full-length form (100%) and to that of the 32-residue truncated form, N*Δ*32, (0%).

Then we set out to determine which part of the N-terminal extension is essential for MOB1 autoinhibition. To this end, a series of N-truncated MOB1B proteins were generated for binding assays to measure reduction in autoinhibition with deletion (Fig. 5a). Interestingly, truncation of the N-terminal 10 residues resulted in 66% relative autoinhibition compared with that of the full-length form, suggesting that formation of the β-sheet between the S_N_ and S2 strands contributes to stabilization of the autoinhibited form (Fig. 5b). Truncation of the N-terminal 16 and 21 residues removes the N-terminal S_N_ strand and the linker, but not the Switch helix, and resulted in a large reduction in autoinhibition (10–20% relative autoinhibition). The peptide region targeted for the truncation contains basic residues (4 Lys residues), which may contribute to the autoinhibition by electrostatically interacting with the negatively-charged LATS1-binding surface of the MOB1 core domain. These results are essentially consistent with a recent report detailing truncation of the N-terminal 15 or 32 residues^27^.

### Structure of the N-truncated MOB1B-LATS1 complex

**Figure 6:**
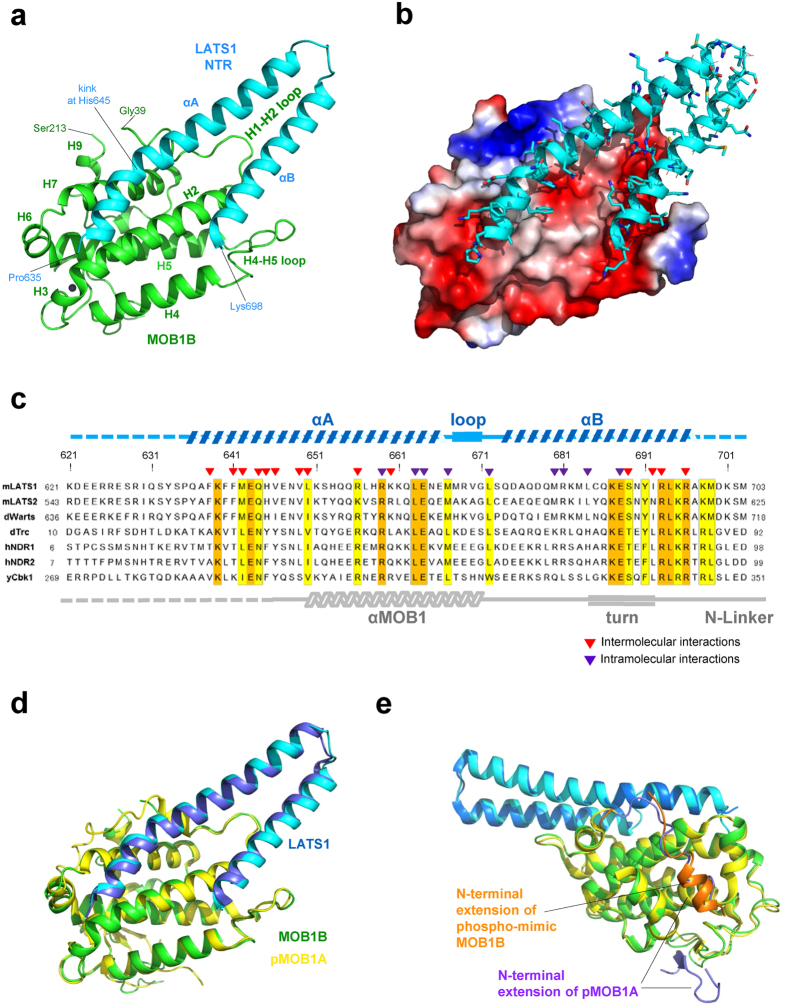
Structure of the MOB1B-LATS1 complex. (**a**) Structure of the complex between N-truncated MOB1B (residues 33-216, green) and the NTR domain (621-703, cyan) of LATS1. The view point is nearly the same as that of full-length MOB1B shown in Fig. 1b. (**b**) As in **a**, but MOB1B is shown as an electrostatic surface potential and the LATS NTR domains shown in a ribbon model with side chains as stick models. (**c**) Sequence alignment of the NTR domains of LATS and NDR kinases. The secondary structure elements of our structure are shown at the top with broken lines for residues undefined in the current electron density map. Two helices contain conserved (orange) and semi-conserved (yellow) residues. Both polar and nonpolar residues participate in intermolecular interactions (red arrow heads) with MOB1B, and intramolecular interactions (purple arrow heads) that stabilize the NTR bihelical structure. The N-terminal 14 residues and a short C-terminal segment comprising 5 residues are unstructured (broken lines). (**d**) Overlay of the structure of the N-truncated MOB1B-LATS1 complex (MOB1B in green and the NTR domain of LATS1 in cyan) on that of the pMOB1A-LATS1 complex (5BRK, MOB1A in yellow and the NTR domain of LATS1 in blue). (**e**) Overlay of the structure of the phosphomimetic MOB1B-LATS1 complex (MOB1B in green and the NTR domain of LATS1 in cyan) on that of the pMOB1A-LATS1 complex (5BRK, as in **d**).

As discussed later, we found a serious discrepancy between the reported structures of the pMOB1A-LATS1 complex^27^ and the Mob2p-Cbk1p complex^26^. We determined the structure of the complex between the N-truncated dominant active form of MOB1B in complex with the NTR domain of LATS1 to independently ascertain which structure of the MOB1-LATS1 complex is correct. The crystal structure of the complex between the NTR (residues 621-703) domain and N-terminal truncated MOB1B (33-216) was determined at 2.96 Å resolution (Table 1). The structure reveals that the NTR domain forms a V-shaped bihelical structure formed by antiparallel long helices, αA (Gln636-Met668) and αB (Gln674-Ala697), which bind the negatively-charged and nonpolar patched surface of MOB1B (Fig. 6a–c). The V shape of the NTR domain is stabilized by formation of an inter-helical hydrophobic core with nonpolar residues (Leu663 and Met667 from the α1 helix, Leu672 from the connected loop, and Met680 and Leu684 from the α2 helix) and inter-helical salt bridges comprising residues, Arg659-Glu688 and Glu664-Arg681 (Supplementary Fig. 4a,b). The obtained structure is essentially the same as that of the pMOB1A-LATS1 complex, with small overall root mean square (r.m.s.) deviation (0.79 Å) (Fig. 6d). Furthermore, our complex structure between the phosphomimetic form of MOB1B bound to the LATS1 NTR domain also displays a similar structure to that of the pMOB1A-LATS1 complex (Fig. 6e).

The major intermolecular interaction between MOB1B and the NTR domain occurs primarily at the sidechain level with both nonpolar and polar contacts but also includes direct interactions involving the backbone of MOB1B (Supplementary Fig. 5). The helical axis of the αA helix is kinked at His645 (Fig. 6a) with the following part of the αA helix docked into the hydrophobic groove between antiparallel helices H2 and H7, and the αB helix interacts with the negatively-charged patch (Fig. 6b and Supplementary Fig. 4c,d). Four negatively-charged acidic residues (Glu49 and Glu51 from the H1-H2 loop, and Glu55 and Asp63 from the H2 helix) of MOB1B form direct salt bridge/hydrogen bond formations with six positively-charged residues (His645, Arg656, Arg659 and Lys660 from the α1 helix, and Arg693 and Arg696 from the α2 helix) from the NTR domain (Supplementary Fig. 6). The observed MOB1B-LATS1 interactions in our structure were verified with mutational analysis. Alanine mutants of highly conserved arginine residues (R656A, R693A and R696A) displayed decreased binding affinity to less than 20% of the wild-type (Supplementary Fig. 7). The S689E mutation, a phosphomimetic substitution, drastically decreased the affinity (to less than 10%). Mutation of both R659 and E688, which do not participate in direct interaction with MOB1B but stabilize the V-shaped structure by forming a salt bridge (Supplementary Fig. 4b), resulted in only a moderate reduction in the affinity (~40%). These mutational analyses are consistent with our complex structure and relevant experimental data from the literature, both with the *in vivo* and *in vitro* binding and kinase assays investigating mutations in human NDR1^30^ and LATS1^32^,^33^. Thus, our structural data verify the reported structure of the pMOB1A-LATS1 complex and significantly expands our understanding of Hippo core signalling on the molecular level.

### Comparison with the Mob2p-Cbk1p complex

**Figure 7:**
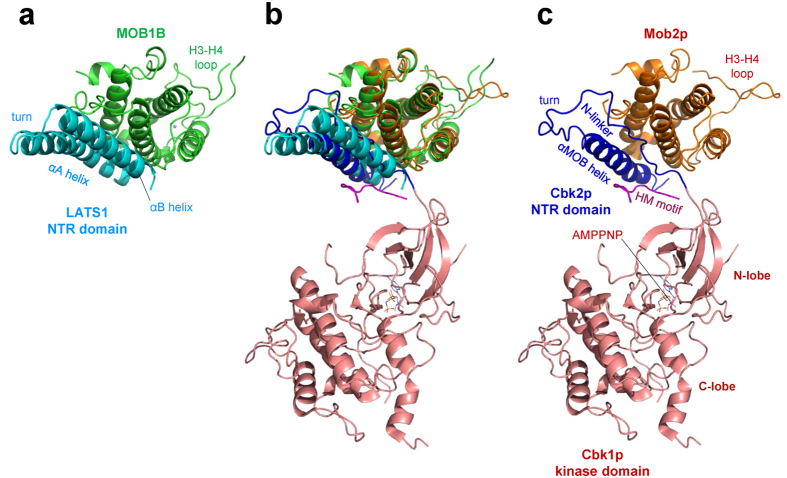
Structural comparison of the MOB1B-LATS1 complex and the Mob2p-Cbk1p complex. (**a**) The MOB1B-LATS1 complex. The NTR domain (cyan) of LATS1 bound to MOB1B (green) with labels for the α1 and α2 helices and the turn between the two helices. (**b**) The superimposed structures shown in (**a,c)**. (**c**) The Mob1p-Cbk1p complex (PDB code 4LQS, at 3.3 Å resolution)^36^. The NTR domain (blue) of Cbk1p bound to Mob1p (orange) consists of a MOB1 helix, a turn and an N-linker which has no regular secondary structure. The kinase domain model lacks the N-lobe αC helix that is invisible in the map.

We then performed a detailed comparison of our complex with the recently reported structure of the Mob2p-Cbk1p complexes^36^ in detail (Fig. 7). Superposition of the MOB1B and Mob2p structures showed fundamental similarity (r.m.s. deviaton of 1.45 Å), although a large local deviation was found in the H3-H4 loop conformation, which may have resulted from absence of two cysteine residues responsible for zinc ion coordination in Mob2p (Supplementary Fig. 3). In stark contrast to the overall similarity of the MOB structures, the NTR domain of Cbk1p displays an unexpectedly different conformation from the bihelical V-shaped structure of the LATS1 NTR domain. The Cbk1p NTR domain forms a shorter α-helix, the αMOB helix, but lacks most of helical structures, including the α2 helix. Although the αMOB helix shows partial overlap with the N-terminal half of the LATS1 α1 helix, the residues assigned to this helix were shifted by 14 residues toward the C-terminus in the aligned sequences of the NTR domains (Fig. 6c, bottom). As a result of these differences, the Mob2p-Cbk1p interactions are completely distinct from those identified in our MOB1B-LATS1 and the pMOB1A-LATS1 complexes. We found that the Mob2p-Cbk1p interactions are inconsistent with relevant experimental data from the literature and our mutation studies. For example, Cbk1p residues (Ser337, Arg341 and Arg344) corresponding to conserved residues (Ser689, Arg693 and Arg696 of LATS1) participating in direct interactions with MOB1B, are located far from the MOB interface. Curiously, the peptide-chain tracing of the Cbk1p NTR domain roughly matches that of our LATS1 NTR domain, implying mis-tracing of the peptide chain of the Cbk1p NTR domain. Further structural studies are needed to clarify whether the structural discrepancies reflect any intrinsic differences between mouse MOB1B and yeast Mob2p structures, differences between a NTR domain fragment and a full-length kinase or any other technical differences in the structural analyses.

## Discussion

**Figure 8:**
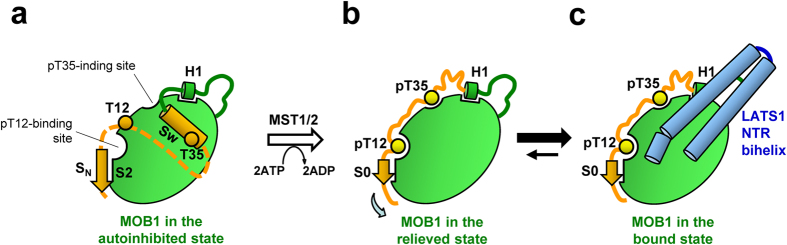
A model of autoinhibition and activation of MOB1. (**a**) The autoinhibited form of MOB1. The N-terminal extension forms the very N-terminal β-strand, the S_N_ strand (residues 5-9), forming a β-sheet with the S2 strand, followed by a conformationally flexible linker (broken line) and the Switch helix (Sw) bound to the LATS1-binding site. The linker between the S_N_ strand and the Switch helix may run at the LATS1-binding surface. Two key threonine residues, Thr12 and Thr35, are phosphorylated by MST1/2 on activation. Thr12 is located at the conformationally flexible linker and Thr35 is located at the Switch helix. (**b**) The phosphorylation-medicated active form of MOB1. Autoinhibition is relieved by unfolding of the Switch helix, which is induced by the “pull-the-string” mechanism with steric effects following phosphorylation of Thr35 and Thr12 and the binding of phosphorylated Thr12 and Thr35 to pT12and pT35 binding pockets, respectively. The β-sheet between the S0 (residues 12-15) and S2 strands replaces that between the S_N_ and S2 strands. (**c**) The V-shaped α-helices of the NTR domain of LATS1 bind the LATS1 binding surface and autoinhibition is relieved by phosphorylation of the N-terminal extension that blocked the LATS1-binding surface of the MOB core domain.

In the present study, we have elucidated the mechanism of MOB1 autoinhibition mediated by the N-terminal extension by structurally blocking the LATS1-binding site, and proposed that autoinhibition is relieved by phosphorylation which enhances dissociation of the N-terminal extension (Fig. 8). In the autoinhibited form of MOB1, the N-terminal extension forms the very N-terminal β-strand, the S_N_ strand (residues 5-9), forming a β-sheet with the S2 strand, followed by a conformationally flexible linker and the Switch helix bound to the LATS1-binding site. The flexible linker runs along the LATS1-binding surface with electrostatic interactions. Thr12 is located at the conformationally flexible linker and Thr35 is located at the Switch helix. The phosphorylation-mediated activation of MOB1 involves dissociation of the Switch helix, which is induced by the “pull-the-string” mechanism with steric effects following the phosphorylation of Thr35 and the binding of phosphorylated Thr12 and Thr35 to pT12 and pT35 binding pockets, respectively. The β-sheet between the S0 (residues 12-15) and S2 strands replaces that between the S_N_ and S2 strands. Interestingly, Thr35 is important in tissue specific processes as inferred from the tissue specific phosphorylation of Thr35^38^.

Formation of the β-sheet between the S_N_ and S2 strands seems to be a key intramolecular interaction that contributes to the autoinhibition of LATS1 binding by the N-terminal extension. Phosphorylation of Thr12 by MST2 interferes with this interaction and results in formation of a β-sheet between the S0 and S2 strands by phosphate-binding to the basic residue cluster pocket. MOB1 is also activated by the phosphorylated peptide region of MST2, which forms a β-sheet with the S2 strand by competition with the S_N_ strand of autoinhibited MOB1^37^. This binding may play a role in formation of a ternary MST1-MOB1-LATS1 complex to accelerate phosphorylation of both MOB1 and LATS1, as previously suggested^37^.

It is noticeable that the N-terminal 20 residues of MOB1 are highly positively charged with basic residues, one Arg and four Lys residues and no acidic residues. Our data suggested that this peptide region is conformationally flexible but critical for the MOB1 autoinhibition, probably mediated by electrostatic interaction with the negatively-charged LATS1-binding surface of MOB1. We speculate that formation of a β-sheet between the S_N_ and S2 strands contributes to autoinhibition by suppressing the conformationally dynamic properties of the flexible N-terminal extension, and stabilizes association of the N-terminal basic region to the negatively-charged LATS1-bindig surface of MOB1. These electrostatic interactions should stabilize association of the Switch helix with the LATS1-binding surface. This may account for the fact that removal of these basic residues by N-terminal truncation resulted in acceleration of LATS1 binding. We speculate that the S_N_ strand, the basic region and the Switch helix of MOB1 cooperatively contribute to autoinhibition by binding to each part of the MOB core domain.

Previous reports have suggested that Thr74 of MOB1 acts as a minor phosphorylation site^23^,^39^. In our structures, Thr74 is located at the C-terminal end of the H2 helix and in the MOB1B-LATS1 complex forms an intramolecular hydrogen bond with Glu175 (H7 helix), while its side-chain methyl group contacts the N-terminal end of the α1 helix of the NTR domain. In yeast, Mob1p is phosphorylated and regulated by the highly conserved Cdk1 kinase, whereas the major phosphorylation sites (Ser36 and Thr85) are located at the yeast-specific N-terminal extension^40^, suggesting that this crosstalk might be specific to yeast cells.

Contrary to the importance of phosphorylation in MOB1 for binding to LATS/NDR kinases, MOB2 does not conserve Thr12/Thr35 residues despite the high sequence homology of its core domain with MOB1 (Supplementary Fig. 3). Moreover, MOB2 exhibits strong binding to NDR1/2 kinases but not to LATS1/2 kinases^33^,^35^. In the MOB1-LATS1 complex structure, three Glu residues (at positions of 49, 51 and 55 of MOB1B) and Asp63 are required for direct interactions with LATS1. Among these negatively-charged key residues, Glu49 and Glu51 are not conserved in MOB2 proteins: these Glu residues are replaced with positively-charged, non-charged or hydrophobic residues (Arg, Lys, Gln, Ile and Val in Supplementary Fig. 3). Moreover, Asp63 is replaced by a Thr residue in human, mouse and xenopus MOB2 proteins. These replacements could account, at least in part, for the failure of MOB2 to bind LATS1/2 kinases.

The binding of MOB2 to NDR kinases, however, could not be directly explained by an examination of our structure, due to the lack of key residues (Glu49, Glu51 and Asp63 of MOB1) required for direct interactions with LATS1 as mentioned above, whereas the binding of MOB1 to NDR kinases is well supported by our complex structure, which showed that key residues of LATS1 required for direct interactions with MOB1 are well conserved in NDR kinases (Fig. 6c). MOB2 binding to NDR1/2 kinases inactivates these kinases by blocking MOB1 binding necessary for stimulating phosphorylation of the A-loop and the hydrophobic motif 35. We speculate that MOB2 binding to NDR kinases differs somewhat from that of MOB1 binding, even though the binding site of MOB2 is mostly or partly overlapped with that of MOB1. This hypothesis is supported by the NDR1 alanine mutations (Tyr31, Arg41, Arg44, Thr74 and Arg78), which had no effect on MOB2 binding but abolished MOB1 binding^35^, since these NDR1 residues correspond to key LATS1 residues (Val646, Arg656, Arg659, Thr689 and Arg693, respectively) required for interactions with the NTR domain. Further structural studies, however, are needed to elucidate details of the MOB2 function.

In our structure, Ser689 of the LATS1 NTR domain is involved in direct interactions with MOB1B (Supplementary Figs 5 and 6b) and the S689E mutation, a phosphomimetic substitution, almost abolished MOB1B binding (Supplementary Fig. 7). However, the Ser689-equivalent residue of NDR1, Thr74, was reported as a putative phosphorylation site and phosphorylation is crucial for interaction with S100B^40^. On the other hand, the reported NMR structure of the S100B-NDR1 complex showed that Thr74 is not located at the S100B-NDR1 interface but is exposed to the solvent region^41^. Therefore, although the mechanism involving enhancement of S100B-NDR1 binding *via* Thr74 phosphorylation remains unknown, these results imply that activation of NDR kinases by S100B binding should differ from that involving MOB1.

Having understood the manner by which MOB1 can be regulated as a result of our study and previous structural work, another area that needs to be addressed is the manner by which MOB1 structurally regulates LATS/NDR kinase activities. MOB1 binding to NDR1/2 kinases triggers auto-phosphorylation of NDR1/2 on the A-loop, and also facilitates phosphorylation of the hydrophobic motif by MST kinases^30^,^31^,^34^,^35^,^43^,^44^. In contrast, MOB1 binding to LATS1/2 kinases is only required for phosphorylation of the A-loop, while phosphorylation of the hydrophobic motif can be uncoupled from MOB1-LATS complex formation^8^,^9^. These facts suggest that LATS1/2 kinase activation by MOB1 should differ in some respect from that of NDR1/2 kinase activations, even though both are mediated by phosphorylation of the A-loop. The recently-reported structures of the Mob2p-Cbk1p complex showed that the NTR domain-bound Mob2p has no direct contact with the kinase catalytic domain, and is located at some distance far from the A-loop at the active site^36^ (Fig. 7c). Furthermore, the N-lobe displays a disordered structure, which lacks helices αB and αC, indicating that the structure should be that of an inactive form, as seen in the reported structure of Akt/PKB in the inactive OFF state^45^. Thus, the precise mechanism by which MOB1 accelerates A-loop phosphorylation remains unknown.

In conclusion, our results have defined MOB1 autoinhibition involving the N-terminal extension containing the Switch helix, basic region and the S_N_ strand, and also revealed a “pull-the-string” mechanism by which Thr12/Thr35 phosphorylation activates MOB1. These findings provide a physical basis for understanding the Hippo pathway.

## Materials and Methods

### Cloning of MOB1B and LATS1 constructs

The full-length cDNA clone of mouse MOB1B (GeneBank code NM026735) and LATS1 (GeneBank code BC158123) were purchased from Open Biosystems. The genes were subcloned into pGEX 6p-3 (GE healthcare) for N-terminal truncated MOB1B (residues 33-216), and pET49b (Merck) or pGEX 6p-3 for full-length MOB1B (1-216). The plasmids were transformed into *E. coli* strain BL21-CodonPlus RIL (Stratagene) or *Rosetta2 (DE3) (Merck)* for protein expression. Cloning regions of LATS1 were decided based on secondary structure prediction using the program Jpred 3^46^. DNA fragments of LATS1 (coding residues 604-703, and 621-703) were subcloned into pET47b *(Merck)* by polymerase chain reaction (PCR) amplification of the cDNA clone as a template. The fidelity of the coding region of all clones was confirmed by DNA sequencing.

### Protein expression and purification

MOB1B proteins were expressed in *E. coli* hosts which were grown in LB medium supplemented with antibiotics at 37 °C to an OD_600_ of 0.6. Expression was induced by the addition of 100 μM isopropyl-β-D-thiogalactoside (IPTG) and 50 μM zinc acetate. Following incubation for another 20 hours at 18 °C, cells were harvested by centrifugation. For the purification of full-length MOB1B, cells were suspended in 2 × PBS (phosphate-buffered saline), containing 1 mM dithiothreitol (DTT), and then disrupted by sonication in an ice bath. The insoluble fraction was removed by centrifugation and the supernatant was loaded onto a Glutathione Sepharose 4B affinity column (GE Healthcare). The column was washed with 3×PBS containing 1 mM DTT, and then the protein was collected using an elution buffer containing 20 mM Tris-HCl (pH 7.4), 0.1 M NaCl, 1 mM DTT and 20 mM glutathione. The column effluent containing GST-MOB1B was digested for 24 hours at 4 °C with HRV 3C protease (Merck). The resultant product was loaded onto a HiTrap Q column (GE Healthcare) for GST-tag removal. The flow-through fractions were pooled and concentrated using Amicon Ultra centrifugal filter devices (10,000 MWCO, Millipore). Protein was finally purified through a Superdex 75pg gel filtration column (GE Healthcare) equilibrated with buffer (20 mM Tris-HCl (pH 7.5), 0.15M NaCl, and 1 mM DTT). Protease inhibitors were not employed during the purification. Double-mutant (T12A/T35A) MOB1B was purified by the same procedure. N-terminal truncated MOB1B was also purified by the same procedure except that HiTrap Q column elution was effected using a 0.1–0.5 M NaCl gradient in buffer containing 25 mM Tris-HCl (pH 8.0) and 1 mM DTT.

For expression of the NTR domains (residues 604-703 and 621-703) of LATS1, plasmids were transformed into BL21 (DE3) Star™ (Invitrogen) which was grown in LB medium supplemented with antibiotics at 37 °C to an OD_600_ of 0.6. Protein expression was induced by the addition of 500 μM of IPTG followed by incubation for another 4 hours at 38 °C. Harvested cells were suspended in lysis buffer containing 20 mM Tris-HCl (pH 8.0), 0.5 M NaCl, 10 mM imidazole, and 3 mM β-mercaptoethanol, and then disruped by sonication in an ice bath. Most of the LATS1 polypeptide was found in the insoluble fraction following centrifugation at 4 °C. For resolubilization of the polypeptide, the pellet was emulsified in PBS supplemented with 3 mM β-mercaptoethanol, and then shaken vigorously for 12 hours at 42 °C. The solubilized polypeptide was clarified by ultracentrifugation and the supernatant was loaded onto a Ni-NTA agarose affinity column (Qiagen). The column was washed with lysis buffer, and protein was eluted using buffer comprising 10 mM Tris-HCl (pH 8.0), 0.1 M NaCl, 3 mM β-mercaptoethanol, and 250 mM imidazole. Protein was further purified using HiTrap SP cation-exchange (GE Healthcare) and Superdex 75pg gel filtration columns. Protein purity was monitored during purification using polyacrylamide gel electrophoresis in the presence of sodium dodecyl sulfate (SDS-PAGE). Purified proteins were concentrated and their molecular weight confirmed by matrix-assisted laser desorption/ionization time-of-flight mass spectrometry (MALDI-TOF MS; Bruker Daltonics). Pull-down binding assays showed that both fragments specifically bound MOB1B (Supplementary Fig. 5).

### Crystallization and data collection

Initial screening of the crystallization conditions was performed by the sitting-drop vapor diffusion method at 4 °C and 20 °C using Mosquito (TTP Labtech) and commercial crystallization kits (Qiagen, Sigma and Hampton Research). Crystallization conditions were then optimized by the hanging drop vapor diffusion method with streak seeding to obtain single crystals suitable for X-ray diffraction. The optimized crystallization conditions for full-length MOB1B included a mixture of 1 μl protein solution (1.8 mM protein in the A buffer) and an equal volume of reservoir solution containing 10–13% PEG 3350, 0.2 M ammonium fluoride, and 0.1 M MES (pH 6.5). Crystals were flash-frozen to 100 K in cryoprotectant solution containing 33–35% (v/v) glycerol.

The best crystal of the MOB1B (33-216) and LATS1 (621-703) complex was obtained by mixing 1 μl protein solution (1.1 mM 1:1 molar ratio mixture in a stock buffer containing 10 mM Tris-HCl (pH 7.5), 0.1 M NaCl, and 0.25 mM TCEP) and 1 μl reservoir solution containing 3–5% PEG 4000 and 0.1 M Na-MES (pH 6.5). Crystals of the double-mutant T12D/T35D MOB1B (1-216) and LATS1 (621-703) complex were obtained by mixing 1 μl protein solution (0.8 mM 1:1 molar ratio mixture in a stock buffer containing 10 mM Tris-HCl (pH 8.0), 0.1 M NaCl, and 0.25 mM TCEP) and 1 μl reservoir solution containing 20% PEG 3350, 0.2 mM Sodium sulfate and 0.1 M MES (pH 6.5). We obtained diamond-shaped crystals of full-length MOB1B and needle crystals of the complexes (Supplementary Fig. 8). X-ray diffraction data were collected on beamlines BL41XU and BL44XU at SPring-8, Japan, and then indexed and merged using the HKL2000 program suite^47^. Crystallographic data are summarized in Table 1.

### Structure determination and refinement

Phases of the MOB1B-LATS1 complex crystal structure were determined by molecular replacement using the program PHASER^48^ with the model of MOB1A (PDB code 1PI1)^8^ as a search model. An initial model equivalent to LATS1 was constructed using the PHENIX Autobuild program^49^ and built manually with reference to the electron density map using Coot^50^. The model was refined using CNS^51^ and PHENIX, and manual adjustments were made using Coot. The refined MOB1B (33-216) model was used as a molecular replacement for determining the phases of the full-length free MOB1B crystal structure. The initial model of the N-terminal region was built manually. Structural refinements were then performed by the same methods used for the complex. The crystal contains four crystallographically independent molecules A-D, whose structures are essentially the same (Supplementary S3b). Two (molecules A and C) of the four molecules display a β-sheet between the S_N_ and S2 strands (see text). Using the refined MOB1B-LATS1 complex, the structure of the double-mutant MOB1B-LATS1 complex was determined and refined. Superposition of the MOB molecules was performed using the program LSQKAB^52^. Illustrations were prepared using the program PyMOL (DeLano Scientific).

### Preparation of mutant proteins and GST pull-down assays

Mutant proteins were generated by PCR site-directed mutagenesis. PCR was performed using wild-type plasmids as a template, and the fidelity of the products was confirmed by DNA sequencing. Mutant proteins were expressed using the same procedure employed for the wild-type. GST-MOB1B (wild-type and double-mutant T12D/T35D full-length forms) were purified using Glutathione Sepharose 4B affinity and HiTrap Q anion-exchange columns. LATS1 mutant polypeptides were resolubilized using the same method as described for the wild-type, and then subjected to further purification using Ni-NTA affinity and Superdex 75pg gel filtration columns. Purified proteins were concentrated, frozen in liquid nitrogen, and then kept at −80 °C until use. To verify the MOB1B-LATS1 interactions, several point mutants of the NTR domain (621-703) were generated. However, we found that some of these mutants could not be utilized due to solubility issue. We therefore used mutant proteins of the NTR domain (604-703). The MOB1B-binding affinity did not differ between the two NTR constructs. For the pull-down assay, GST-MOB1B (the N-terminal 32-residue truncated form) and LATS1 proteins (40 μM) were mixed in 1:1 molar ratio in 200 μl assay buffer containing 20 mM Tris-HCl (pH 7.5), 0.3 M NaCl and 1 mM DTT, and then applied to 100 μl Glutathione Sepharose 4B resin equilibrated with the same buffer. The resin was washed three times with 700 μl assay buffer and centrifugation (500 g for 5 min) at 4 °C, followed by verification of the supernatant OD_280_ at a basal level. Resin-attached proteins were then eluted using 150 μl assay buffer supplemented with 30 mM glutathione, and the effluents analyzed by 15% SDS-PAGE. The intensities of the bands stained by Coomassie blue were visualized using ImageQuant LAS-4000 (Fujifilm). The bars in Figs 4b and 5b show relative intensities analyzed using the program ImageGauge (Fujifilm).

### Analytical ultracentrifugation (AUC)

Sedimentation velocity ultracentrifugation experiments were performed at 20 °C using a Beckman Coulter Optima XLA analytical ultracentrifuge equipped with an An-60 Ti rotor and double-sector centerpieces as previously described^53^. Protein samples adjusted to 19 μM in buffer containing 20 mM Tris-HCl (pH 7.5) and 0.1 M NaCl were centrifuged at 20,000 rpm (32,198 g) and then scanned at a wavelength of 280 nm. Radial absorbance scans were measured 99 times every 12 minutes at a wavelength of 280 nm. For high protein concentration analysis, MOB1B adjusted to 200 μM in the same buffer was scanned at 300 nm for saturation issue. The resultant data were analyzed using the program SEDFIT.

## Additional Information

**Accession Codes**: Atomic coordinates and structural factors for the full-length MOB1B, the MOB1B-LATS1 complex and the doubly phosphomimic (T12D/T35D) MOB1B-LATS1 complex have been deposited in the Protein Data Bank under ID code 5B5V, 5B5W and 5B6B, respectively.

**How to cite this article**: Kim, S.-Y. *et al*. Structural basis for autoinhibition and its relief of MOB1 in the Hippo pathway. *Sci. Rep.* **6**, 28488; doi: 10.1038/srep28488 (2016).

## Supplementary Material

Supplementary Information

## Figures and Tables

**Table 1:** Crystallographic and refinement statistics.

	Full-length MOB1B^a^	MOB1B-LATS1^a^	MOB1B(T12/35D)-LATS1^a^
Data collection			
Space group	*P*2_1_	*C*2_1_	*C*222_1_
Cell parameters [Å]	*a* = 59.36, *b* = 127.69, *c* = 59.30	*a* = 78.75, *b* = 71.76, *c* = 57.86	*a* = 168.65, *b* = 301.44, *c* = 127.38
*V*_M_ [Å^3^/Da]/*V*_sol_ [%]	2.22/44.71	2.29/46.39	2.84/56.71
SPring-8 beam line	BL44XU	BL41XU	BL44XU
Resolution [Å]^b^	30-2.20 (2.24-2.20)	30-2.96 (3.00-2.96)	50-3.55 (3.61-3.55)
Wavelength [Å]	0.9000	1.0000	0.9000
Reflections (unique)	159,366 (44,345)	19,931 (6,660)	297,262 (39,931)
Completeness [%]	99.3 (100)	94.1 (82.5)	100 (100)
Redundancy	3.6 (3.8)	3.2 (3.1)	7.5 (7.6)
Mean *I*/σ_*I*_	37.7 (3.5)	21.4 (2.3)	21.0 (3.8)
*R*_merge_ [%]	6.3 (55.4)	7.4 (56.5)	15.3 (72.4)
Refinement			
No. atoms			
Protein	6,323	1420/546 (MOB1/LATS1)	11,855/4,750
Zn^2+^ ion	4	1	8
Cl^−^ ion	2	–	7
Solvent	153	–	–
*R*_work_/*R*_free_ [%]^c^	23.3/24.6	23.4/27.0	21.6/26.2
Ramachandran plot			
Favored [%]	96.4	92.6	95.7
Allowed [%]	3.6	6.5	3.3
Outliers [%]	0	0.9	1
r. m. s. deviations			
Bond lengths [Å]	0.013	0.003	0.003
Bond angles [°]	1.323	0.745	0.754
Average B-factor [Å^2^]			
Protein	58.8	103.0/98.9	101.9
Zn^2+^ ion	60.0	107.4	135.8
Cl^−^ ion	69.7	–	57.1
Solvent	57.6	–	–

^a^One crystal was used for each data set. The total oscillation range was 180°.

^b^Numbers in parentheses refer to statistics for the outer resolution shell.

^c^*R*_work_ = Σ| | *F*_obs_ | − | *F*_calc_ | |/Σ| *F*_obs_ |. *R*_free_ is the same as *R*_work_ except that a 5% subset of all reflections was held aside throughout the refinement.

## References

[b1] Edgar B. A. From cell structure to transcription: Hippo forges a new path. Cell 124, 267–73 (2006). 16439203 10.1016/j.cell.2006.01.005

[b2] Saucedo L. J. & Edgar B. A. Filling out the Hippo pathway. Nat Rev Mol Cell Biol 8, 613–21 (2007). 17622252 10.1038/nrm2221

[b3] Zhao B., Tumaneng K. & Guan K. L. The Hippo pathway in organ size control, tissue regeneration and stem cell self-renewal. Nat Cell Biol 13, 877–83 (2011). 21808241 10.1038/ncb2303 PMC3987945

[b4] Ramos A. & Camargo F. D. The Hippo signaling pathway and stem cell biology. Trends Cell Biol 22, 339–46 (2012). 22658639 10.1016/j.tcb.2012.04.006 PMC3383919

[b5] Harvey K. F., Zhang X. & Thomas D. M. The Hippo pathway and human cancer. Nat Rev Cancer 13, 246–57 (2013). 23467301 10.1038/nrc3458

[b6] Johnson R. & Halder G. The two faces of Hippo: targeting the Hippo pathway for regenerative medicine and cancer treatment. Nat Rev Drug Discov 13, 63–79 (2014). 24336504 10.1038/nrd4161 PMC4167640

[b7] Chan E. H. . The Ste20-like kinase Mst2 activates the human large tumor suppressor kinase Lats1. Oncogene 24, 2076–2086 (2005). 15688006 10.1038/sj.onc.1208445

[b8] Praskova M., Xia F. & Avruch J. MOBKL1A/MOBKL1B phosphorylation by MST1 and MST2 inhibits cell proliferation. Curr Biol 18, 311–21 (2008). 18328708 10.1016/j.cub.2008.02.006 PMC4682548

[b9] Hoa L. . The characterisation of LATS2 kinase regulation in Hippo-YAP signalling. Cell Signal 28, 488–497 (2016). 26898830 10.1016/j.cellsig.2016.02.012

[b10] Huse M. & Kuriyan J. The conformational plasticity of protein kinases. Cell 109, 275–82 (2002). 12015977 10.1016/s0092-8674(02)00741-9

[b11] Yin F. . Spatial Organization of Hippo Signaling at the Plasma Membrane Mediated by the Tumor Suppressor Merlin/NF2. Cell 154, 1342–1355 (2013). 24012335 10.1016/j.cell.2013.08.025 PMC3835333

[b12] Lignitto L. . Proteolysis of MOB1 by the ubiquitin ligase praja2 attenuates Hippo signalling and supports glioblastoma growth. Nat Commun 4 (2013). 10.1038/ncomms2791 PMC3674242 23652010

[b13] Kim M. . cAMP/PKA signalling reinforces the LATS-YAP pathway to fully suppress YAP in response to actin cytoskeletal changes. Embo Journal 32, 1543–1555 (2013). 23644383 10.1038/emboj.2013.102 PMC3671250

[b14] Serrano I., McDonald P. C., Lock F., Muller W. J. & Dedhar S. Inactivation of the Hippo tumour suppressor pathway by integrin-linked kinase. Nat Commun 4, 2976 (2013). 24356468 10.1038/ncomms3976 PMC3905719

[b15] Bae S. J. . NEDD4 controls intestinal stem cell homeostasis by regulating the Hippo signalling pathway. Nat Commun 6, 6314 (2015). 25692647 10.1038/ncomms7314

[b16] Luca F. C. & Winey M. MOB1, an essential yeast gene required for completion of mitosis and maintenance of ploidy. Mol Biol Cell 9, 29–46 (1998). 9436989 10.1091/mbc.9.1.29 PMC25214

[b17] Mah A. S., Jang J. & Deshaies R. J. Protein kinase Cdc15 activates the Dbf2-Mob1 kinase complex. Proc Natl Acad Sci USA 98, 7325–30 (2001). 11404483 10.1073/pnas.141098998 PMC34667

[b18] Lai Z. C. . Control of cell proliferation and apoptosis by mob as tumor suppressor, mats. Cell 120, 675–685 (2005). 15766530 10.1016/j.cell.2004.12.036

[b19] Wei X., Shimizu T. & Lai Z. C. Mob as tumor suppressor is activated by Hippo kinase for growth inhibition in Drosophila. Embo Journal 26, 1772–1781 (2007). 17347649 10.1038/sj.emboj.7601630 PMC1847660

[b20] Hergovich A., Stegert M. R., Schmitz D. & Hemmings B. A. NDR kinases regulate essential cell processes from yeast to humans. Nat Rev Mol Cell Biol 7, 253–64 (2006). 16607288 10.1038/nrm1891

[b21] Nelson B. . RAM: A conserved signaling network that regulates Ace2p transcriptional activity and polarized morphogenesis. Mol Biol Cell 14, 3782–3803 (2003). 12972564 10.1091/mbc.E03-01-0018 PMC196567

[b22] Jansen J. M., Barry M. F., Yoo C. K. & Weiss E. L. Phosphoregulation of Cbk1 is critical for RAM network control of transcription and morphogenesis. J Cell Biol 175, 755–766 (2006). 17145962 10.1083/jcb.200604107 PMC2064675

[b23] Bao Y. . Roles of mammalian sterile 20-like kinase 2-dependent phosphorylations of Mps one binder 1B in the activation of nuclear Dbf2-related kinases. Genes Cells 14, 1369–81 (2009). 19919647 10.1111/j.1365-2443.2009.01354.x

[b24] Stavridi E. S. . Crystal structure of a human Mob1 protein: toward understanding Mob-regulated cell cycle pathways. Structure 11, 1163–70 (2003). 12962634 10.1016/s0969-2126(03)00182-5

[b25] Ponchon L., Dumas C., Kajava A. V., Fesquet D. & Padilla A. NMR solution structure of Mob1, a mitotic exit network protein and its interaction with an NDR kinase peptide. J Mol Biol 337, 167–82 (2004). 15001360 10.1016/j.jmb.2004.01.010

[b26] Mrkobrada S., Boucher L., Ceccarelli D. F., Tyers M. & Sicheri F. Structural and functional analysis of Saccharomyces cerevisiae Mob1. J Mol Biol 362, 430–40 (2006). 16934835 10.1016/j.jmb.2006.07.007

[b27] Rock J. M. . Activation of the yeast Hippo pathway by phosphorylation-dependent assembly of signaling complexes. Science 340, 871–5 (2013). 23579499 10.1126/science.1235822 PMC3884217

[b28] Chung H. Y., Gu M., Buehler E., MacDonald M. R. & Rice C. M. Seed sequence-matched controls reveal limitations of small interfering RNA knockdown in functional and structural studies of hepatitis C virus NS5A-MOBKL1B interaction. J Virol 88, 11022–33 (2014). 25031347 10.1128/JVI.01582-14 PMC4178819

[b29] Zhou D. . Mst1 and Mst2 maintain hepatocyte quiescence and suppress hepatocellular carcinoma development through inactivation of the Yap1 oncogene. Cancer Cell 16, 425–38 (2009). 19878874 10.1016/j.ccr.2009.09.026 PMC3023165

[b30] Bichsel S. J., Tamaskovic R., Stegert M. R. & Hemmings B. A. Mechanism of activation of NDR (nuclear Dbf2-related) protein kinase by the hMOB1 protein. J Biol Chem 279, 35228–35 (2004). 15197186 10.1074/jbc.M404542200

[b31] Hergovich A., Bichsel S. J. & Hemmings B. A. Human NDR kinases are rapidly activated by MOB proteins through recruitment to the plasma membrane and phosphorylation. Mol Cell Biol 25, 8259–72 (2005). 16135814 10.1128/MCB.25.18.8259-8272.2005 PMC1234321

[b32] Bothos J., Tuttle R. L., Ottey M., Luca F. C. & Halazonetis T. D. Human LATS1 is a mitotic exit network kinase. Cancer Research 65, 6568–6575 (2005). 16061636 10.1158/0008-5472.CAN-05-0862

[b33] Hergovich A., Schmitz D. & Hemmings B. A. The human tumour suppressor LATS1 is activated by human MOB1 at the membrane. Biochem Biophys Res Commun 345, 50–58 (2006). 16674920 10.1016/j.bbrc.2006.03.244

[b34] Hergovich A. . The MST1 and hMOB1 tumor suppressors control human centrosome duplication by regulating NDR kinase phosphorylation. Curr Biol 19, 1692–702 (2009). 19836237 10.1016/j.cub.2009.09.020

[b35] Kohler R. S., Schmitz D., Cornils H., Hemmings B. A. & Hergovich A. Differential NDR/LATS interactions with the human MOB family reveal a negative role for human MOB2 in the regulation of human NDR kinases. Mol Cell Biol 30, 4507–20 (2010). 20624913 10.1128/MCB.00150-10 PMC2937529

[b36] Gogl G. . The Structure of an NDR/LATS Kinase-Mob Complex Reveals a Novel Kinase-Coactivator System and Substrate Docking Mechanism. PLos Biol 13, e1002146 (2015). 25966461 10.1371/journal.pbio.1002146 PMC4428629

[b37] Ni L., Zheng Y., Hara M., Pan D. & Luo X. Structural basis for Mob1-dependent activation of the core Mst-Lats kinase cascade in Hippo signaling. Genes Dev. 29, 1416–1431 (2015). 26108669 10.1101/gad.264929.115 PMC4511216

[b38] Huttlin E. L. . A tissue-specific atlas of mouse protein phosphorylation and expression. Cell 143, 1174–89 (2010). 21183079 10.1016/j.cell.2010.12.001 PMC3035969

[b39] Hirabayashi S. . Threonine 74 of MOB1 is a putative key phosphorylation site by MST2 to form the scaffold to activate nuclear Dbf2-related kinase 1. Oncogene 27, 4281–4292 (2008). 18362890 10.1038/onc.2008.66

[b40] König C., Maekawa H. & Schiebel E. Mutual regulation of cyclin-dependent kinase and the mitotic exit network. J Cell Biol 188, 351–68 (2010). 20123997 10.1083/jcb.200911128 PMC2819678

[b41] Tamaskovic R., Bichsel S. J., Rogniaux H., Stegert M. R. & Hemmings B. A. Mechanism of Ca2+-mediated regulation of NDR protein kinase through autophosphorylation and phosphorylation by an upstream kinase. J Biol Chem 278, 6710–6718 (2003). 12493777 10.1074/jbc.M210590200

[b42] Bhattacharya S., Large E., Heizmann C. W., Hemmings B. & Chazin W. J. Structure of the Ca2+/S100B/NDR kinase peptide complex: Insights into S100 target specificity and activation of the kinase. Biochemistry 42, 14416–14426 (2003). 14661952 10.1021/bi035089a

[b43] Stegert M. R., Hergovich A., Tamaskovic R., Bichsel S. J. & Hemmings B. A. Regulation of NDR protein kinase by hydrophobic motif phosphorylation mediated by the mammalian Ste20-like kinase MST3. Mol Cell Biol 25, 11019–29 (2005). 16314523 10.1128/MCB.25.24.11019-11029.2005 PMC1316964

[b44] Vichalkovski A. . NDR kinase is activated by RASSF1A/MST1 in response to Fas receptor stimulation and promotes apoptosis. Curr Biol 18, 1889–95 (2008). 19062280 10.1016/j.cub.2008.10.060

[b45] Yang J. . Crystal structure of an activated Akt/protein kinase B ternary complex with GSK3-peptide and AMP-PNP. Nat Struct Biol 9, 940–4 (2002). 12434148 10.1038/nsb870

[b46] Cole C., Barber J. D. & Barton G. J. The Jpred 3 secondary structure prediction server. Nucleic Acids Res 36, W197–201 (2008). 18463136 10.1093/nar/gkn238 PMC2447793

[b47] Otwinowski Z. & Minor W. Processing of X-ray diffraction data collected in oscillation mode. Macromolecular Crystallography, Pt A 276, 307–326 (1997). 10.1016/S0076-6879(97)76066-X 27754618

[b48] Mccoy A. J. . Phaser crystallographic software. J Appl Crystallogr 40, 658–674 (2007). 19461840 10.1107/S0021889807021206 PMC2483472

[b49] Adams P. D. . PHENIX: a comprehensive Python-based system for macromolecular structure solution. Acta Crystallogr D Biol Crystallogr 66, 213–21 (2010). 20124702 10.1107/S0907444909052925 PMC2815670

[b50] Emsley P. & Cowtan K. Coot: model-building tools for molecular graphics. Acta Crystallogr D Biol Crystallogr 60, 2126–32 (2004). 15572765 10.1107/S0907444904019158

[b51] Brunger A. T. . Crystallography & NMR system: A new software suite for macromolecular structure determination. Acta Crystallogr D Biol Crystallogr 54, 905–21 (1998). 9757107 10.1107/s0907444998003254

[b52] Kabsch W. Solution for Best Rotation to Relate 2 Sets of Vectors. Acta Crystallographica Section A 32, 922–923 (1976).

[b53] Mori T., Gotoh S., Shirakawa M. & Hakoshima T. Structural basis of DDB1-and-Cullin 4-associated Factor 1 (DCAF1) recognition by merlin/NF2 and its implication in tumorigenesis by CD44-mediated inhibition of merlin suppression of DCAF1 function. Genes Cells 19, 603–19 (2014). 24912773 10.1111/gtc.12161

