# Supplementary material for: Structural basis for autoinhibition and its relief of MOB1 in the Hippo pathway
Source: Sci Rep. 2016 Jun 23;6:28488. doi: 10.1038/srep28488 (PMC4917820; doi:10.1038/srep28488)
Supplement: Supplementary Information [file srep28488-s1.pdf]

Structural basis for autoinhibition and its relief of MOB1 in the Hippo pathway

Sun-Yong Kim, Yuka Tachioka, Tomoyuki Mori and Toshio Hakoshima*

Structural Biology Laboratory, Nara Institute of Science and Technology, 8916-5 Takayama, Ikoma, Nara 630-0192, Japan

* Corresponding author:
T. Hakoshima, Structural Biology Laboratory, Nara Institute of Science and Technology,
8916-5 Takayama, Ikoma, Nara 630-0192, Japan.
Tel.: +81-743-72-5570; Fax: +81-743-72-5579; E-mail: hakosima@bs.naist.jp

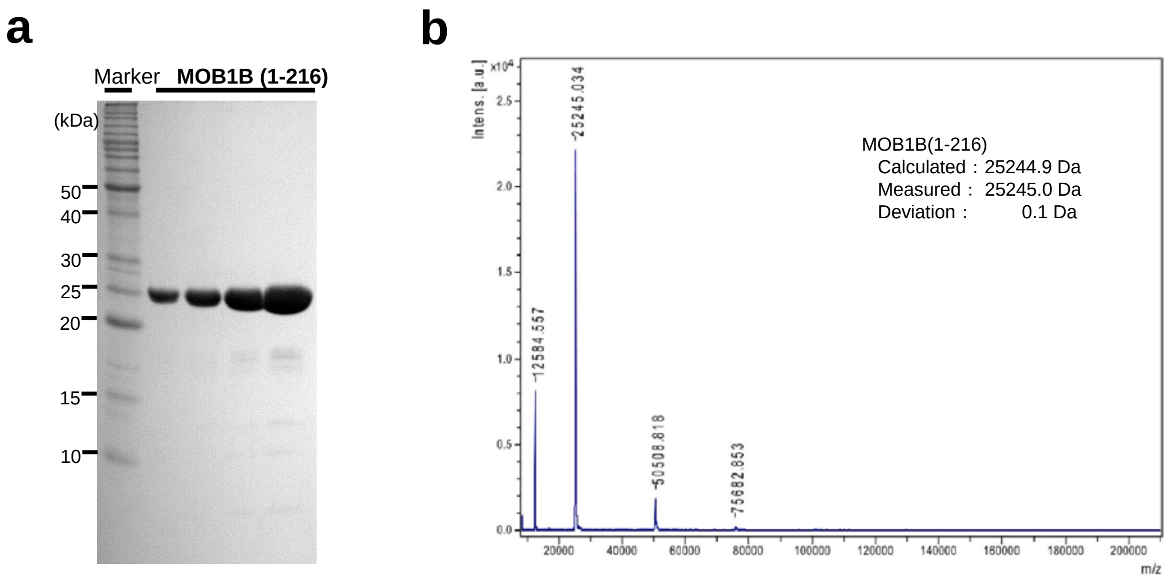

Supplementary Figure 1
Full-length MOB1B was purified without degradation.
(**a**) SDS-PAGE of purified full-length MOB1B. The purified sample gave a single band corresponding to ~25 kDa.
(**b**) MALDI-TOF MS spectrum of purified full-length MOB1B. The spectrum confirmed that the protein had been successfully purified without degradation.

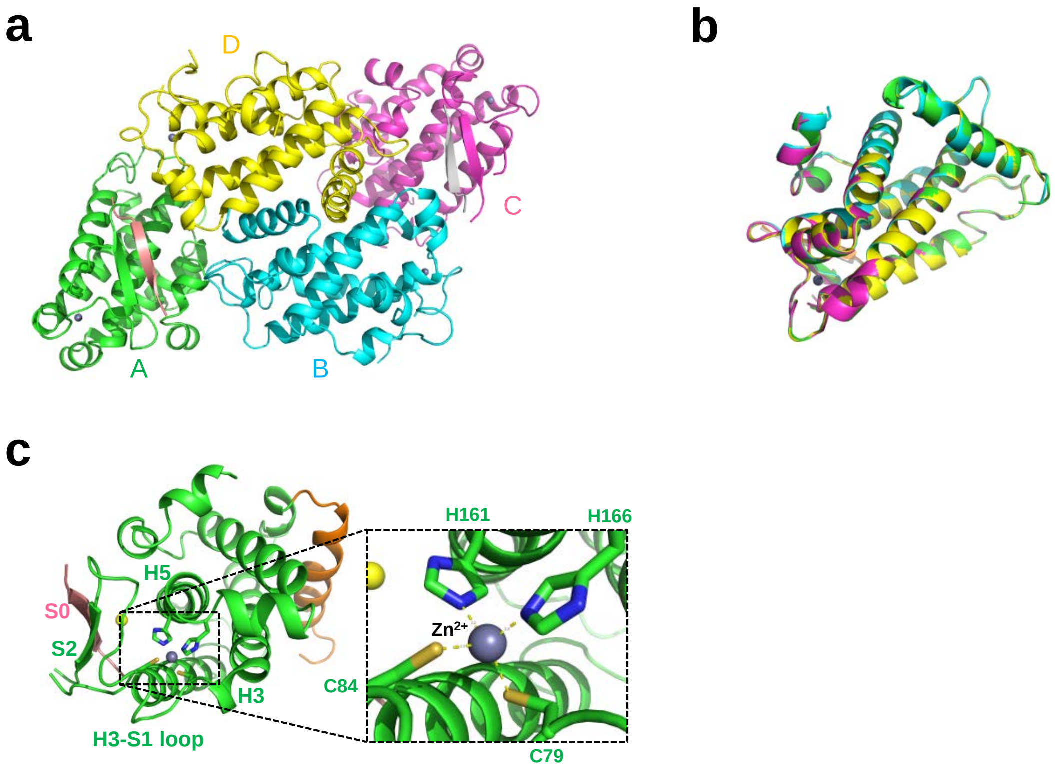

Supplementary Figure 2

Structures of the zinc-, peptide- and chlorine ion-binding sites of full-length MOB1B.

(**a**) Crystal packing of full-length MOB1B. Four crystallographically independent molecules in the asymmetric unit of the crystal are shown in different colors, molecules A (green), B (cyan), C (magenta), and D (yellow). In molecules A and C, the S2 strand binds the S0 strand to form an antiparallel β-sheet.

(**b**) Overlay of four crystallographically independent molecules in the asymmetric unit of the crystal. No significant deviations were found with a small averaged r.m.s. deviation (0.26 Å).

(**c**) The zinc ion-binding site of full-length MOB1B. The zinc ion is coordinated with Cys79 and Cys84 from the H3-S1 loop, and His161 and His166 from the H5 helix.

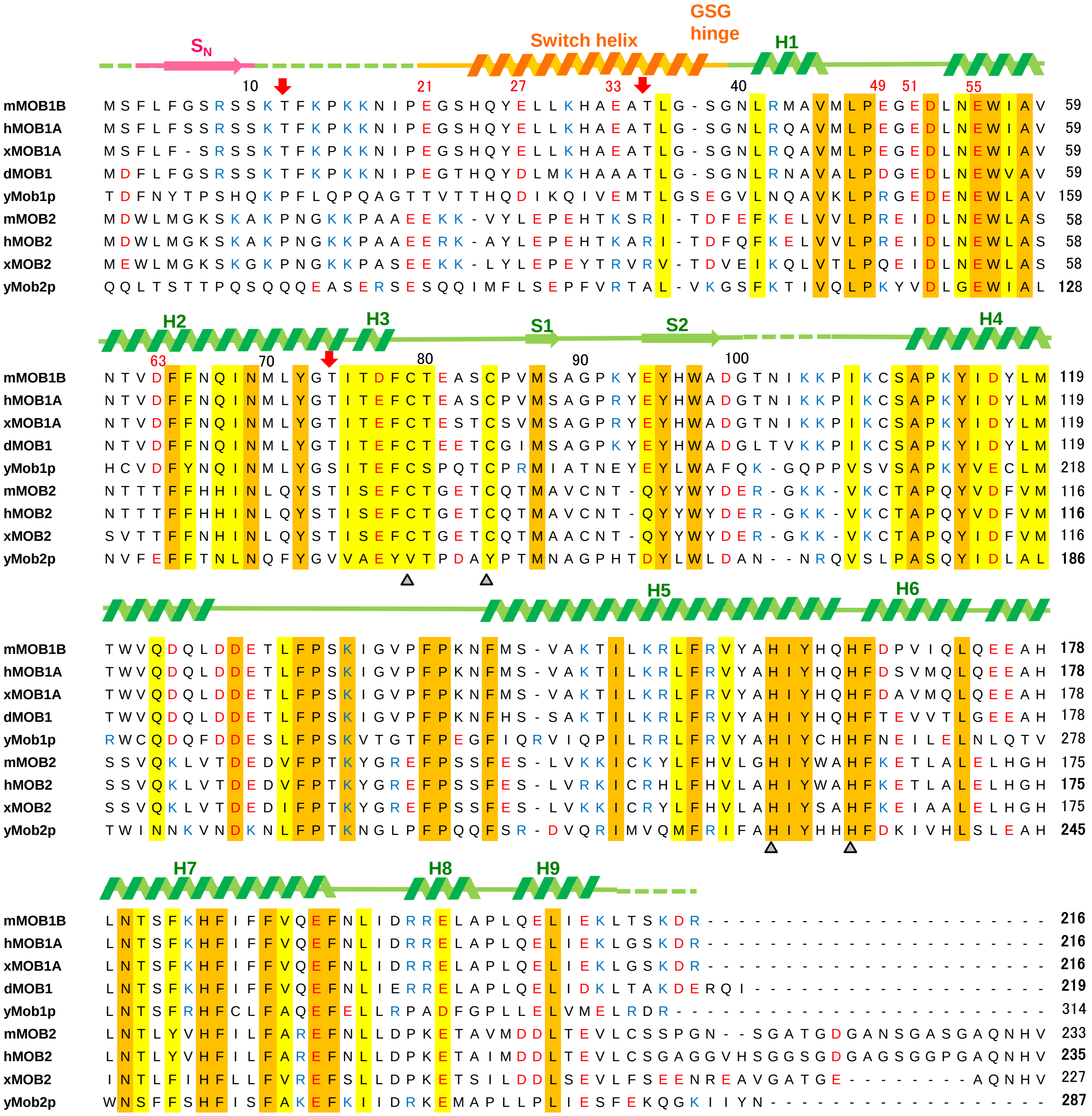

Supplementary Figure 3

Sequence alignment of MOB proteins and the secondary structure elements found in the full-length MOB1B structure of the current study.

The secondary structure elements found in MOB1B structures are shown at the top with α-helices (springs, H1-H9) and β-strands (arrows; S_N, S1 and S2). In our structure, the S1 strand (Met87-Ser88-Ala89) is deformed but forms a type-I β-hairpin with the S2 strand, as in the structure of the N-terminal truncated human MOB1A[24]. MOB sequences are from mouse (m), human (h), *Xenopus* (x), *Drosophila* (d) and yeast (y). Three threonine residues of mouse MOB1B, Thr12, Thr35 and Thr74, are marked with red arrows at the top. Two cysteine and two histidine residues (Cys79, Cys84, His161 and His166 of mouse MOB1B) are marked with grey arrow heads at the bottom. Acidic and basic residues are shown in red and blue, respectively. Conserved residues are highlighted in orange and semi-conserved residues in yellow. Acidic residues interacting with LATS1 are numbered at the top.

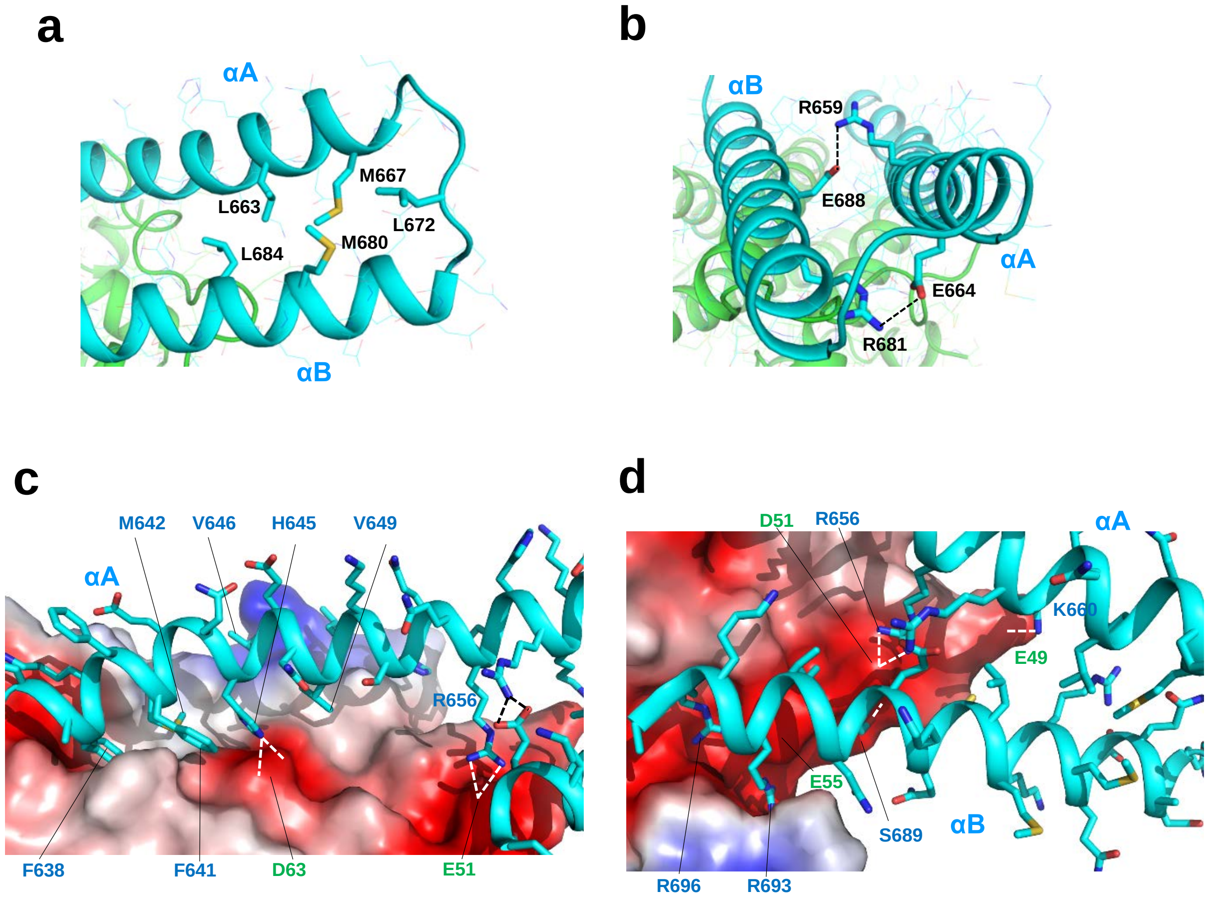

Supplementary Figure 4

Salt bridging/hydrogen bonding interactions between the NTR domain of LATS1 and the negatively-charged nonpolar-patched surface of MOB1B.

(**a**) Close-up view of the inter-helical hydrophobic core formed by nonpolar residues from the αA helix (Leu663 and Met667), the connected loop (Leu672), and the αB helix (Met680 and Leu684).

(**b**) Close-up view of inter-helical salt bridges/hydrogen bonds, Arg596-Glu688 and Glu664-Arg681.

(**c**) Close-up view of the N-terminal region of the αA helix from the NTR domain of LATS1 bound to the groove between helices H1 and H7 of MOB1B. The interactions are shown with broken lines.

(**d**) Close-up view of the helix bundle of the NTR domain of LATS1 bound to the negatively-charged groove between helices H1 and H7 of MOB1B.

a

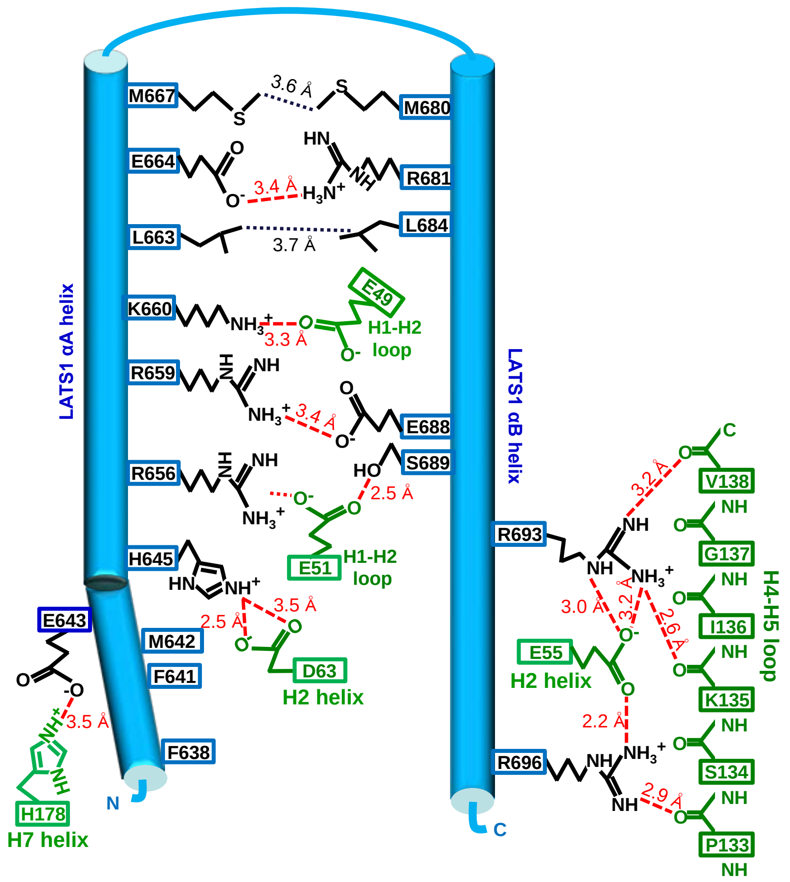

Supplementary Figure 5.

Intramolecular interactions stabilizing the V-shaped structure of the NTR domain of LATS1.

Summary of intermolecular salt bridges/hydrogen bonds between the NTR domain of LATS1 and the MOB core domain of MOB1B (green). A schematic representation of the interactions, dotted lines for nonpolar contacts and red broken lines for hydrogen bonds, with distances is shown.

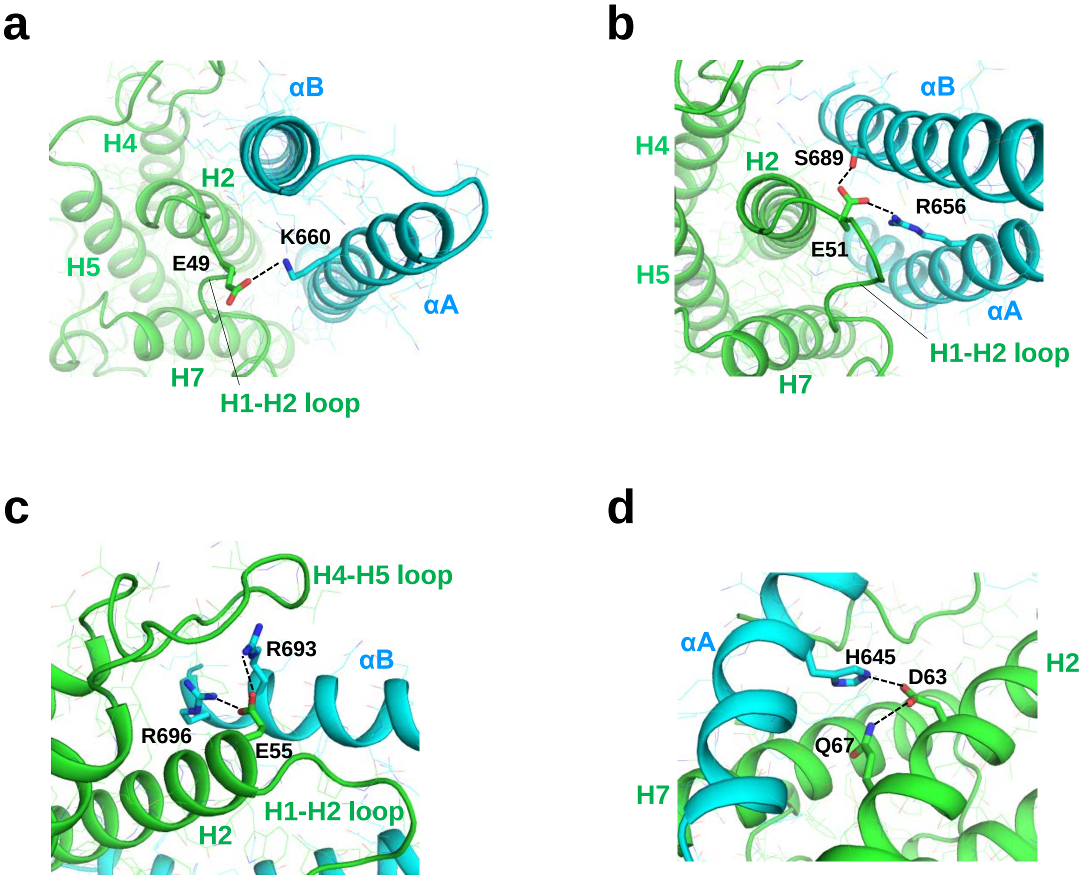

Supplementary Figure 6.

Salt bridging/hydrogen bonding interactions between the NTR domain of LATS1 and the negatively-charged nonpolar-patched surface of MOB1B.

(**a**) Close-up view of the intermolecular salt bridge/hydrogen bond with Glu49 (the H1-H2 loop of MOB1B) and Lys660 (the αA helix of LATS1).

(**b**) Close-up view of the intermolecular salt bridge/hydrogen bond with Glu51 (MOB1B H1-H2 loop) and Arg656 (the αA helix of LATS1) and Ser689 (the αB helix of LATS1).

(**c**) Close-up view of the intermolecular salt bridge/hydrogen bond with Glu55 (the H2 helix of MOB1B) and Arg693 and Arg696 (the αB helix of LATS1).

(**d**) Close-up view of the intermolecular salt bridge/hydrogen bond with Asp63 (the H2 helix of MOB1B) and His645 (the αA helix of LATS1), and Gln67 (MOB1B) stabilizing the Asp63 conformation.

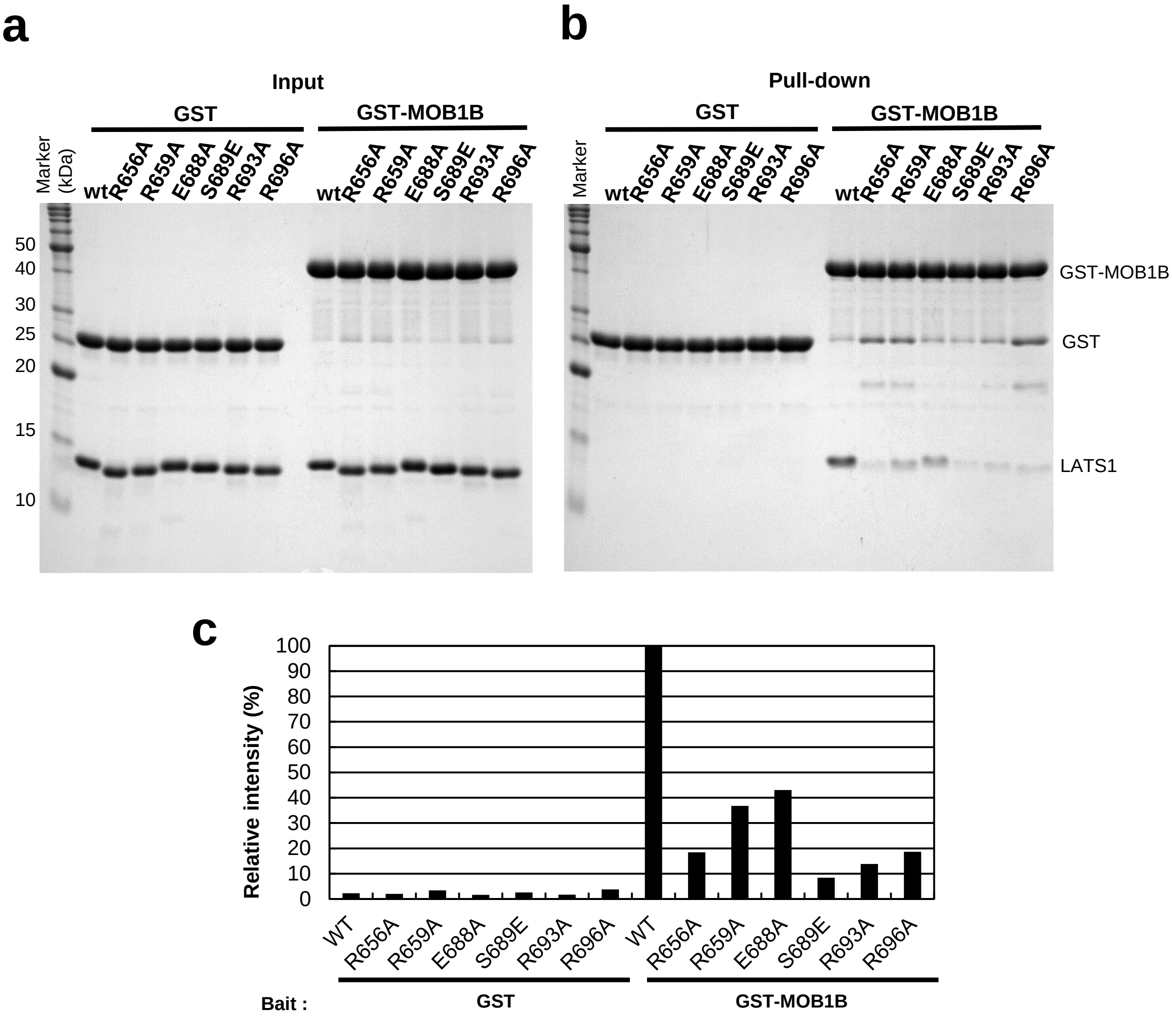

Supplementary Figure 7.

MOB1-LATS1 binding assay with mutations in the NTR domain of LATS1.

(**a**) Input of protein samples in pull-down binding assays of wild-type and mutant NTR domains of LATS1 with GST-MOB1B of the N-truncated form (33-126).

(**b**) Pull-down binding assays of wild-type and mutant NTR domains of LATS1 with GST-N-truncated MOB1B of the N-truncated form (33-126).

(**c**) Histogram of relative LATS1 binding (100% for the wild type) shown in b.

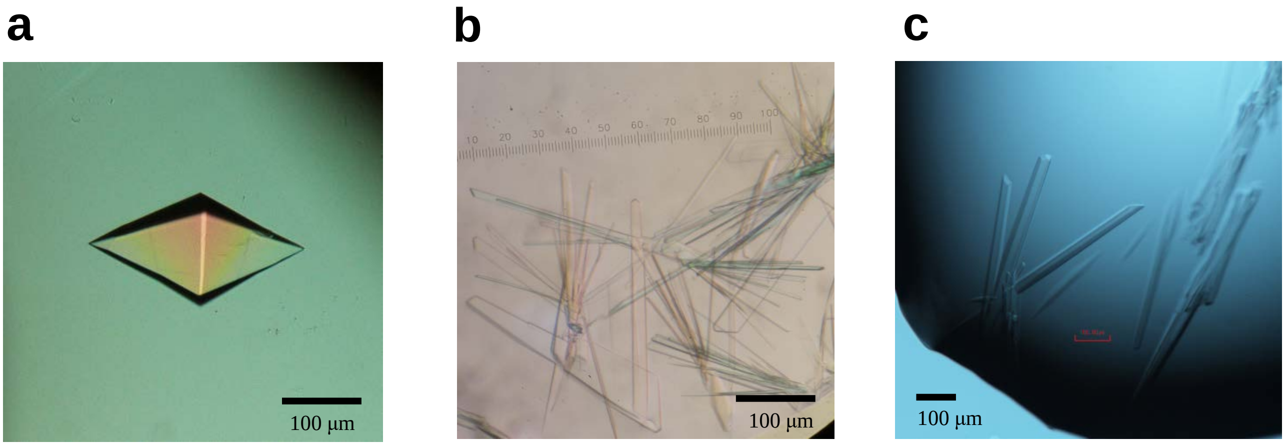

Supplementary Figure 8.

Crystals of full-length MOB1B, the MOB1-LATS1 complex, and the MOB1(T12/35D)-LATS1 complex.

(**a**) Crystal of full-length MOB1B.

(**b**) Crystals of the complex between MOB1B (residues 33-216) and the LATS1 NTR (residues 621-703) domain.

(**c**) Crystals of the complex between MOB1B (T12/35D) and the LATS1 NTR (residues 621-703) domain.
